# Ketamine vs Electroconvulsive Therapy for Treatment-Resistant Depression: A Secondary Analysis of a Randomized Clinical Trial

**DOI:** 10.1001/jamanetworkopen.2024.17786

**Published:** 2024-06-25

**Authors:** Manish Kumar Jha, Samuel T. Wilkinson, Kamini Krishnan, Katherine A. Collins, Gerard Sanacora, James Murrough, Fernando Goes, Murat Altinay, Amy Aloysi, Ali Asghar-Ali, Brian Barnett, Lee Chang, Sara Costi, Donald Malone, Sina Nikayin, Steven E. Nissen, Robert Ostroff, Irving Reti, Kathy Wolski, Dong Wang, Bo Hu, Sanjay J. Mathew, Amit Anand

**Affiliations:** 1 Center for Depression Research and Clinical Care, Department of Psychiatry, The University of Texas Southwestern Medical Center, Dallas; 2 Peter O’Donnell Jr Brain Institute, The University of Texas Southwestern Medical Center, Dallas; 3 Department of Psychiatry, Yale University School of Medicine, New Haven, Connecticut; 4 Lou Ruvo Center for Brain Health, Cleveland Clinic, Cleveland, Ohio; 5 Clinical Research Division, Nathan Kline Institute for Psychiatric Research, Orangeburg, New York; 6 Department of Psychiatry, Icahn School of Medicine at Mount Sinai, New York, New York; 7 Department of Psychiatry and Behavioral Sciences, Johns Hopkins University School of Medicine, Baltimore, Maryland; 8 Department of Psychiatry and Psychology, Center for Behavioral Health, Neurological Institute, Cleveland Clinic, Cleveland, Ohio; 9 Department of Psychiatry and Behavioral Sciences, Baylor College of Medicine, Houston, Texas; 10 Michael E. DeBakey Department of Veterans Affairs Medical Center, Houston, Texas; 11 The Menninger Clinic, Houston, Texas; 12 Department of Anesthesiology, Baylor College of Medicine, Houston, Texas; 13 Psychopharmacology and Emotion Research Laboratory, Department of Psychiatry, University of Oxford, Oxford, United Kingdom; 14 C5Research, Heart, Vascular, and Thoracic Institute, Cleveland Clinic, Cleveland, Ohio; 15 Department of Quantitative Health Sciences, Cleveland Clinic, Lerner Research Institute, Cleveland, Ohio; 16 Department of Psychiatry, Brigham and Women’s Hospital, Harvard Medical School, Boston, Massachusetts

## Abstract

**Importance:**

The ELEKT-D: Electroconvulsive Therapy (ECT) vs Ketamine in Patients With Treatment Resistant Depression (TRD) (ELEKT-D) trial demonstrated noninferiority of intravenous ketamine vs ECT for nonpsychotic TRD. Clinical features that can guide selection of ketamine vs ECT may inform shared decision-making for patients with TRD.

**Objective:**

To evaluate whether selected clinical features were associated with differential improvement with ketamine vs ECT.

**Design, Setting, and Participants:**

This secondary analysis of an open-label noninferiority randomized clinical trial was a multicenter study conducted at 5 US academic medical centers from April 7, 2017, to November 11, 2022. Analyses for this study, which were not prespecified in the trial protocol, were conducted from May 10 to Oct 31, 2023. The study cohort included patients with TRD, aged 21 to 75 years, who were in a current nonpsychotic depressive episode of at least moderate severity and were referred for ECT by their clinicians.

**Exposures:**

Eligible participants were randomized 1:1 to receive either 6 infusions of ketamine or 9 treatments with ECT over 3 weeks.

**Main Outcomes and Measures:**

Association between baseline factors (including 16-item Quick Inventory of Depressive Symptomatology Self-Report [QIDS-SR16], Montgomery-Asberg Depression Rating Scale [MADRS], premorbid intelligence, cognitive function, history of attempted suicide, and inpatient vs outpatient status) and treatment response were assessed with repeated measures mixed-effects model analyses.

**Results:**

Among the 365 participants included in this study (mean [SD] age, 46.0 [14.5] years; 191 [52.3%] female), 195 were randomized to the ketamine group and 170 to the ECT group. In repeated measures mixed-effects models using depression levels over 3 weeks and after false discovery rate adjustment, participants with a baseline QIDS-SR16 score of 20 or less (−7.7 vs −5.6 points) and those starting treatment as outpatients (−8.4 vs −6.2 points) reported greater reduction in the QIDS-SR16 with ketamine vs ECT. Conversely, those with a baseline QIDS-SR16 score of more than 20 (ie, very severe depression) and starting treatment as inpatients reported greater reduction in the QIDS-SR16 earlier in course of treatment (−8.4 vs −6.7 points) with ECT, but scores were similar in both groups at the end-of-treatment visit (−9.0 vs −9.9 points). In the ECT group only, participants with higher scores on measures of premorbid intelligence (−14.0 vs −11.2 points) and with a comorbid posttraumatic stress disorder diagnosis (−16.6 vs −12.0 points) reported greater reduction in the MADRS score. Those with impaired memory recall had greater reduction in MADRS during the second week of treatment (−13.4 vs −9.6 points), but the levels of MADRS were similar to those with unimpaired recall at the end-of-treatment visit (−14.3 vs −12.2 points). Other results were not significant after false discovery rate adjustment.

**Conclusions and Relevance:**

In this secondary analysis of the ELEKT-D randomized clinical trial of ECT vs ketamine, greater improvement in depression was observed with intravenous ketamine among outpatients with nonpsychotic TRD who had moderately severe or severe depression, suggesting that these patients may consider ketamine over ECT for TRD.

## Key Points

**Question:**

Are baseline clinical features associated with differential improvement with ketamine vs electroconvulsive therapy (ECT) in adults with treatment-resistant depression?

**Findings:**

In this secondary analysis of a randomized clinical trial, 365 adults with nonpsychotic treatment-resistant major depression, moderately severe or severe pretreatment depression severity and initiating treatment as an outpatient were associated with greater improvement with ketamine vs ECT. Very severe pretreatment depression severity was associated with greater reduction in self-reported depression severity with ECT vs ketamine earlier during the treatment, but the scores were similar by the end-of-treatment visit.

**Meaning:**

These results suggest that outpatients and those with moderately severe or severe depression may consider ketamine over ECT for treatment-resistant depression.

## Teaser

This secondary analysis of a randomized clinical trial assesses whether baseline clinical features may be associated with differential improvement with ketamine vs electroconvulsive therapy in adults with nonpsychotic treatment-resistant major depression.

## Introduction

Up to 1 in 3 adults with major depressive disorder (MDD) may have treatment-resistant depression (TRD), as they do not experience adequate improvement with 2 or more treatment courses with antidepressants.^1^ Patients with TRD have greater illness burden and higher rates of intentional self-harm and all-cause mortality compared with other patients with MDD.^2,3^ Fewer than 1 in 5 patients with TRD attains remission (ie, experiences no to minimal symptoms) with commonly used antidepressants or their combinations.^1^ Therefore, they may need interventions such as electroconvulsive therapy (ECT),^4^ considered one of the most effective approaches for TRD.^5^ Racemic ketamine, a dissociative anesthetic medication, is also used for TRD,^6,7^ and an intranasally administered (*S*)-enantiomer was approved by the US Food and Drug Administration for this indication in 2019.^8^ To compare an acute course of intravenous racemic ketamine with ECT, the ELEKT-D: Electroconvulsive Therapy (ECT) vs Ketamine in Patients With Treatment-Resistant Depression (TRD) (ELEKT-D) trial enrolled 403 patients with nonpsychotic TRD across 5 sites in the US (Baylor College of Medicine, Cleveland Clinic, Icahn School of Medicine at Mount Sinai, Johns Hopkins University, and Yale University).^9^ As reported previously by Anand et al,^10^ rates of response (≥50% reduction in the 16-item Quick Inventory of Depressive Symptomatology Self-Report [QIDS-SR16, in which scores range from 0 to 27, with >20 indicating very severe depression] at the end-of-treatment visit) with ketamine (55.4%) were noninferior to ECT (41.2%). However, there is decisional uncertainty for patients with TRD and clinicians when selecting between ketamine and ECT. Therefore, identifying baseline (ie, pretreatment) features that may be associated with differential improvement with ketamine vs ECT may be helpful in shared decision-making approaches for patients with TRD. The prespecified subgroup analyses for heterogeneity of the treatment response in the ELEKT-D trial examined limited features, including mean (SD) age (46.0 [14.5] years), sex, self-reported race (Black, White, and other [including American Indian or Native American, Asian, multiracial, and other self-reported races], self-reported ethnicity [Hispanic and non-Hispanic]), admission status at first treatment (inpatient or outpatient), comorbid generalized anxiety disorder, study site, and subtype of depression (melancholic or nonmelancholic), and found no significant interactions with treatment group regarding treatment response.^10^ This secondary analysis of the ELEKT-D randomized clinical trial was designed to further explore factors that may be associated with treatment improvement.

## Methods

### Study Design

Detailed methods of the ELEKT-D trial were published previously,^9,10^ and the study protocol was previously reported by Anand et al^10^ and included as supplemental material (Supplement 1) in this study. The ELEKT-D trial was registered at ClinicalTrials.gov (ClinicalTrials.gov Identifier: NCT03113968). At each site, approval from the institutional review board was obtained prior to participant enrollment. At 5 academic sites, from April 7, 2017, to November 11, 2022, patients with TRD who were referred for ECT by their clinicians were invited to participate in the trial and were enrolled after obtaining written informed consent from each participant. Participants of the study were 21 to 75 years of age and met the *Diagnostic and Statistical Manual of Mental Disorders* (Fifth Edition) criteria for MDD without psychotic features in a current depressive episode lasting at least 4 weeks that was at least moderately severe (a score of >20, according to the Montgomery-Åsberg Depression Rating Scale [MADRS], a 10-item clinician-rated scale, ranging from 0 to 60, with scores >36 indicating very severe depression, designed to detect changes due to antidepressant treatment^11^). Furthermore, participants had a Young Mania Rating Scale score of 5 or less (in which scores range from 0 to 60, with higher scores indicating severe manic symptoms), a Montreal Cognitive Assessment (MoCA) score of 18 or more (in which scores range from 0 to 30, with higher scores indicating normal cognition), and a lifetime history of an unsatisfactory response to at least 2 adequate antidepressant trials. Key exclusion criteria included diagnosis of bipolar disorder, schizophrenia, schizophreniform disorder, schizoaffective disorder, intellectual disability, or pervasive developmental disorder; any contraindications for clinical use of ECT or ketamine treatment based on clinical guidelines or investigator judgement; pregnancy or breastfeeding; severe medical illness or neurological disorders; known ketamine allergy or treated with a medication that may interact with ketamine; and MDD with psychotic features during the current episode. This study followed the Consolidated Standards of Reporting TRIALS (CONSORT) reporting guideline.

Eligible participants were randomized using a secure electronic data-management system in a 1:1 fashion after stratification by site for open-label treatment with either ECT or ketamine for 3 weeks. Those randomized to ketamine received twice-weekly infusions over 3 weeks (a total of 6 infusions). Each infusion contained a subanesthetic dose of 0.5 mg/kg of body weight and was administered for over 40 minutes with allowance for dose modification if clinically indicated. Those randomized to ECT received 3 treatments per week (a total of 9 treatments over 3 weeks) with the recommended starting procedure as a right unilateral ultrabrief pulse width at 6 times the seizure threshold determined during the titration at the first visit,^4^ with subsequent modifications of settings and electrode placements permitted if clinically indicated. The recommendations for both ketamine and ECT were meant to reflect their clinical use, and discontinuation by participants of study treatments was permitted for any reason. Furthermore, study investigators could also discontinue these treatments early (ie, before the end of the 3-week period) if clinically indicated. These early completers were encouraged to participate in end-of-treatment visits. During treatment with either ECT or ketamine, participants were allowed to continue their previously prescribed medications with changes permitted as clinically indicated. This study was based on a modified intent-to-treat sample and included participants who received either ketamine or ECT and completed at least 1 posttreatment assessment (see eFigure 1 in Supplement 2 for the CONSORT diagram). Analyses for this study, which were not prespecified in the trial protocol, were conducted from May 10 to October 31, 2023.

### Clinical Assessments and Study Outcomes

Race and ethnicity for each participant were self-reported and collected as part of study demographics data. Categories were the same as in the ELEKT-D trial. The QIDS-SR16 was the primary outcome clinical measure.^10^ The total score of the QIDS-SR16 ranges from 0 to 27 and is based on the 9 criterion symptom domains of a major depressive episode, in which each domain is scored from 0 to 3.^12^ The MADRS, designed to detect changes due to antidepressant treatment,^11^ was an additional measure of overall depression severity. A score of more than 20 on the QIDS-SR16 indicates very severe depression and corresponds to the score of more than 36 on the MADRS.^13^ The primary outcome of ELEKT-D was based on the QIDS-SR16 and was defined as a decrease from the baseline (first treatment visit) of at least 50% at the end-of-treatment visit, which occurred within 3 days after the last treatment session. Remission based on the QIDS-SR16 and the MADRS was defined as scores of 5 or less and 10 or less, respectively.

Cognitive tests were administered by trained research personnel supervised by a clinical neuropsychologist (K.K.). The North American Adult Reading Test-35 (NAART-35) was administered once as an estimate of premorbid intelligence.^14^ The MoCA was used with the total educational level corrected score. The delayed recall T score for the Hopkins Verbal Learning Test-Revised (HVLT-R) was used as a memory test,^15^ in which scores range from −11 to 62 in ELEKT-D, with higher scores indicating better functioning. The MoCA and the HVLT-R were administered at baseline and at the end of the treatment. Normative data were derived based on published norms from the developer of each measure where applicable. The binary outcome for NAART-35 was assigned using a standard score of less than 85 (low average or below), indicating scores that were 1.5 SDs or more below published normative data by the developers of NAART-35, in which scores range from 57 to 113 in ELEKT-D, with higher scores indicating higher premorbid intelligence.

### Baseline Features Evaluated for an Association With Differential Improvement With ECT vs Ketamine

Based on existing literature,^16,17,18,19,20,21,22,23,24^ the study team identified the following baseline features to evaluate for associations with differential improvement with ECT vs ketamine: NAART-35, baseline depression severity (either the QIDS-SR16 or the MADRS, based on the estimated outcome), cognitive functioning (total educational level corrected score of the MoCA and the T score on the HVLT-R delayed recall), concurrent use of benzodiazepine or of an atypical antipsychotic medication, obesity (as measured by body mass index [BMI], calculated as weight in kilograms divided by height in meters squared), history of attempted suicide, inpatient vs outpatient status at first treatment, the presence of anxious features based on the *Diagnostic and Statistical Manual of Mental Disorders* (Fifth Edition) specifier, and the presence of a comorbid posttraumatic stress disorder (PTSD) diagnosis.

### Statistical Analysis 

Descriptive statistics were used for baseline features evaluated for associations with differential improvement with ketamine vs ECT. Separate sets of models were used for self-reported (QIDS-SR16) and clinician-rated (MADRS) measures of depression severity. Repeated measures mixed-effects model analyses were used for continuous outcomes (levels of symptom severity at each visit as the dependent variable and visit as the within-participant variable) and logistic regression for dichotomous outcomes (response and remission at the end-of-treatment visit). For all regression analyses, site, age, sex, race, and ethnicity were included as covariates.

To identify whether baseline clinical features can identify individuals who may experience greater improvement with ketamine vs ECT, the baseline feature-by-treatment-by-visit interaction was the independent variable of interest in the mixed-effects model analyses, whereas baseline feature-by-treatment interaction was used as the independent variable of interest in logistic regression analyses. Post hoc interpretation of significant interactions was done by stratifying on the features identified in these regression analyses.

Additional exploratory analyses were conducted after stratification by treatment group to evaluate how these features were associated with improvement among those who initiated treatment either with ketamine or with ECT separately. Therefore, a separate set of repeated measures mixed-effects model analyses was used for the ketamine and ECT groups with depression severity as the dependent variable and baseline feature-by-visit as the independent variable of interest. Similarly, a separate set of logistic regression analyses was used for the ECT and ketamine groups for categorical outcomes (ie, response and remission, based both on the QIDS-SR16 and the MADRS).

False discovery rate (FDR) was calculated using the Benjamini-Hochberg procedure to account for multiple comparisons. However, unadjusted 2-sided *P* values < .05 were also reported given the exploratory nature of these analyses. All analyses were conducted in SAS, version 9.4 (SAS Institute Inc) and RStudio in R, version 4.3.1 (R Project for Statistical Computing).

## Results

Among the 365 participants included in this study (mean [SD] age, 46.0 [14.5] years), 191 (52.3%) were women, 20 (5.5%) were Black, 31 (8.5%) were Hispanic, 319 (81.4%) were White, and 334 (91.5%) were non-Hispanic. Participants included 174 men (47.4%), and 26 (7.1%) who were categorized as other race. There were 195 participants randomized to the ketamine group (53.4%) and 170 to the ECT group (46.6%). Descriptive statistics of clinical and demographic features, including those evaluated for associations with treatment outcomes, are reported in Table 1. Descriptive statistics of these features among ELEKT-D participants randomized to ECT who did not complete any posttreatment assessments (33 of 203 who were randomized to ECT and thus were excluded from this study) vs those who did are presented in eTable 1 in Supplement 2. Among the 200 participants randomized to ketamine, only 5 did not complete any posttreatment assessments and thus were excluded from the study.

**Table 1.** Clinical and Demographic Features of ELEKT-D Participants

Feature	Participants		
	Total (N = 365)	ECT group (n = 170)	Ketamine group (n = 195)
**Categorical variables, No. (%)**			
Sex			
Female	191 (52.3)	87 (51.2)	104 (53.3)
Male	174 (47.7)	83 (48.8)	91 (46.7)
Race			
Black	20 (5.5)	10 (5.9)	10 (5.1)
White	319 (87.4)	151 (88.8)	168 (86.2)
Other^a^	26 (7.1)	9 (5.3)	17 (8.7)
Ethnicity			
Hispanic	31 (8.5)	7 (4.1)	24 (12.3)
Non-Hispanic	334 (91.5)	163 (95.9)	171 (87.7)
History of suicide attempt	142 (38.9)	70 (41.2)	72 (36.9)
Anxious features present	198 (54.2)	90 (52.9)	108 (55.4)
Comorbid PTSD	80 (21.9)	43 (25.3)	37 (19.0)
Concurrent benzodiazepine use	114 (31.2)	56 (32.9)	58 (29.7)
Concurrent atypical antipsychotic medication use	103 (28.2)	47 (27.6)	56 (28.7)
Inpatient at first treatment	44 (12.1)	21 (12.4)	23 (11.8)
Site			
Baylor College of Medicine	82 (22.5)	36 (21.2)	46 (23.6)
Cleveland Clinic	105 (28.8)	51 (30.0)	54 (27.7)
Icahn School of Medicine at Mount Sinai	63 (17.3)	28 (16.5)	35 (17.9)
Yale University	75 (20.5)	37 (21.8)	38 (19.5)
Johns Hopkins University	40 (11.0)	18 (10.6)	22 (11.3)
**Continuous variables, mean (SD)**			
Age, y	46.0 (14.5)	46.6 (14.1)	45.6 (14.8)
QIDS-SR16 score^b^	18.1 (4.1)	18.2 (4.2)	17.9 (4.1)
MADRS score^c^	32.5 (6.1)	32.6 (6.1)	32.4 (6.2)
MoCA score^d^	26.5 (2.7)	26.4 (2.6)	26.5 (2.8)
HVLT-R delayed recall T score^e^	38.5 (14.6)	37.8 (14.7)	39.1 (14.6)
NAART-35 score^f^	88.7 (9.5)	89.4 (8.7)	88.0 (10.2)
BMI	29.9 (7.6)	30.5 (7.9)	29.5 (7.4)

Abbreviations: BMI, body mass index (calculated as weight in kilograms divided by height in meters squared); ECT, electroconvulsive therapy; ELEKT-D, ELEKT-D: Electroconvulsive Therapy (ECT) vs Ketamine in Patients With Treatment Resistant Depression (TRD); HVLT-R, Hopkins Verbal Learning Test-Revised; MADRS, Montgomery-Åsberg Depression Rating Scale; MoCA, Montreal Cognitive Assessment; NAART-35, North American Adult Reading Test-35; PTSD, posttraumatic stress disorder; QIDS-SR16, 16-item Quick Inventory of Depressive Symptomatology Self-Report.

^a^ Included American Indian or Native American, Asian, multiracial, and other races that were self-reported.

^b^ Scores range from 0 to 27, with more than 20 indicating very severe depression.

^c^ A 10-item clinician-rated scale, ranging from 0 to 60, in which a score greater than 36 indicates very severe depression.

^d^ Scores range from 0 to 30, with higher scores indicating normal cognition.

^e^ Scores range from −11 to 62 in ELEKT-D, with higher scores indicating better functioning.

^f^ Scores range from 57 to 113 in ELEKT-D, with higher scores indicating higher premorbid intelligence.

### Baseline Features Associated With Improvement With Ketamine vs ECT

**Figure 1.**
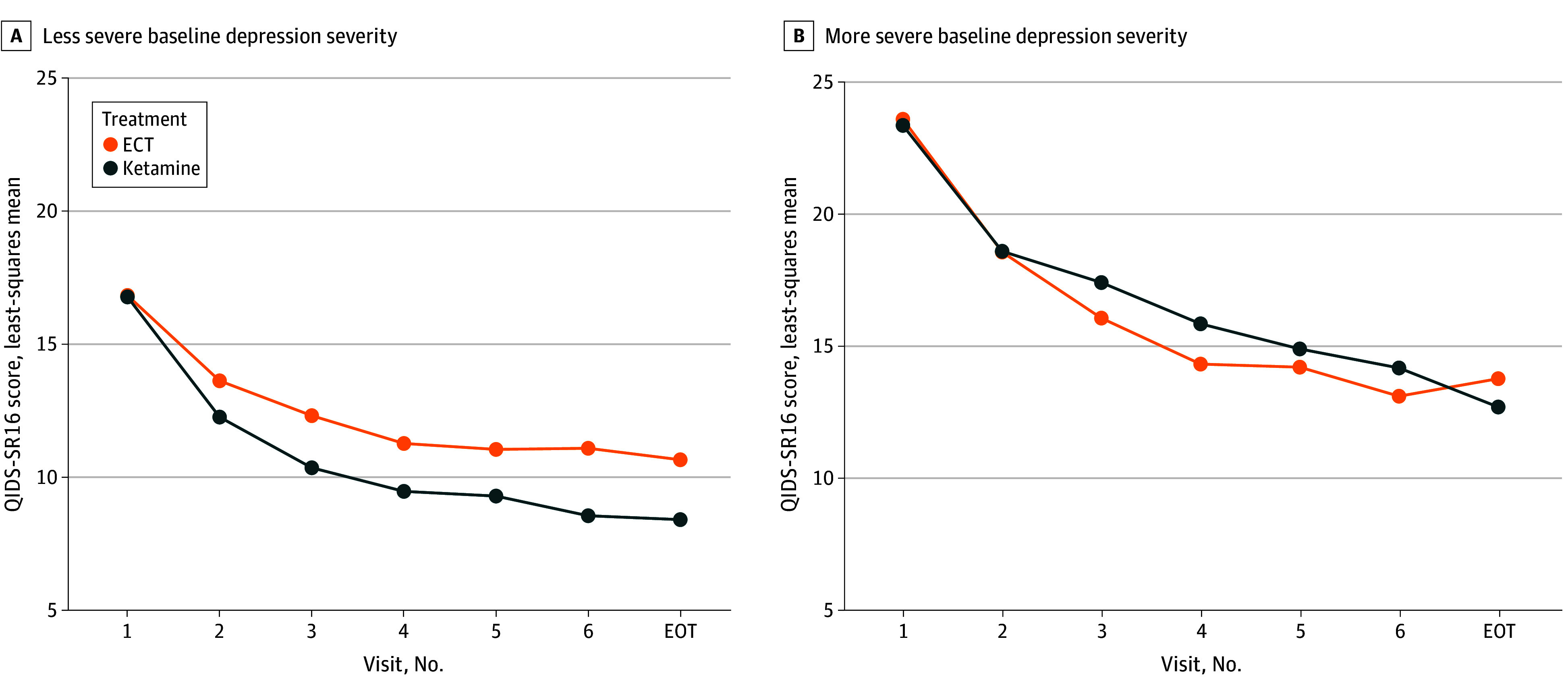
Treatment Outcomes of Ketamine vs Electroconvulsive Therapy (ECT) Stratified by Less or More Severe Baseline Depression Severity The least-squares mean from mixed-effects model analyses was plotted for both treatment groups (ECT and ketamine) based on the 16-item Quick Inventory of Depressive Symptomatology Self-Report (QIDS-SR16) baseline depression severity thresholds of moderate severe or severe (score of ≤20) or very severe (score of >20), in which scores range from 0 to 27, with more than 20 indicating very severe depression. EOT indicates end-of-treatment visit.

After FDR adjustment, baseline QIDS-SR16 and inpatient status at first treatment were significantly associated with improvement in the QIDS-SR16 with ketamine vs ECT (see Table 2 for all results). Participants with moderately severe or severe depression (ie, a QIDS-SR16 score ≤20^13^) at baseline had a greater reduction in the QIDS-SR16 (Figure 1A) with ketamine (−7.7 points) compared with ECT (−5.6 points). Conversely, participants with very severe depression (ie, a QIDS-SR16 score >20) had greater reduction in the QIDS-SR16 with ECT (−8.4 points) vs ketamine (−6.7 points) earlier during treatment (ie, by week 2), but the 2 groups were similar at the end of the 3-week period (−9.0 vs −9.9 points) (Figure 1B). Furthermore, participants initiating treatment as outpatients had greater reduction in the QIDS-SR16 with ketamine vs ECT (−8.4 vs −6.2 points), whereas those initiating treatment as inpatients had greater reduction with ECT vs ketamine (−10.9 vs −8.0 points) (see eFigure 2A in Supplement 2 showing levels of depression severity from baseline to the end-of-treatment visit and eFigure 2B in Supplement 2 showing changes in depression severity from baseline to the end-of-treatment visit). In the ECT group only, participants with higher scores on measures of premorbid intelligence (−14.0 vs −11.2 points) and with a comorbid posttraumatic stress disorder diagnosis (−16.6 vs −12.0 points) reported greater reduction in the MADRS score. Those with impaired memory recall had greater reduction in MADRS during the second week of treatment (−13.4 vs −9.6 points), but the levels of MADRS were similar to those with unimpaired recall at the end-of-treatment visit (−14.3 vs −12.2 points).

**Table 2.** Baseline Features Associated With Differential Improvement in Depression Severity With Ketamine vs Electroconvulsive Therapy

Measure by treatment-by-time interaction	*F* test	*df* ^a^	*P* value
**QIDS-SR16** ^b^			
NAART-35 standardized score^c^	1.38	6/2004	.22
Baseline QIDS-SR16 score^b^	3.19	6/2021	.004^d^
MoCA score^e^	1.22	6/2020	.29
HVLT-R delayed recall T score^f^	0.56	6/2015	.76
Concurrent benzodiazepine use	1.16	6/2020	.33
Concurrent atypical antipsychotic medication use	1.02	6/2021	.41
BMI	1.31	6/1940	.25
History of suicide attempt	0.54	6/2021	.78
Inpatient status at first treatment	3.95	6/2021	<.001^d^
Presence of anxious features	0.50	6/2021	.81
Comorbid PTSD diagnosis	0.66	6/2021	.69
**MADRS** ^g^			
NAART-35 standardized score^c^	2.07	6/2005	.05
Baseline MADRS score^g^	3.03	6/2016	.006
MoCA score^e^	1.27	6/2020	.27
HVLT-R delayed recall T score^f^	1.39	6/2016	.22
Concurrent benzodiazepine use	0.97	6/2021	.44
Concurrent atypical antipsychotic medication use	1.68	6/2022	.12
BMI	1.25	6/1941	.28
History of suicide attempt	0.24	6/2022	.96
Inpatient status at first treatment	1.73	6/2022	.11
Presence of anxious features	0.84	6/2022	.54
Comorbid PTSD diagnosis	0.72	6/2022	.63

Abbreviations: BMI, body mass index (calculated as weight in kilograms divided by height in meters squared); HVLT-R, Hopkins Verbal Learning Test-Revised; MADRS, Montgomery-Åsberg Depression Rating Scale; MoCA, Montreal Cognitive Assessment; NAART-35, North American Adult Reading Test-35; PTSD, posttraumatic stress disorder; QIDS-SR16, 16-item Quick Inventory of Depressive Symptomatology Self-Report.

^a^ Presented as numerator *df*/denominator *df*.

^b^ Scores range from 0 to 27, with more than 20 indicating very severe depression.

^c^ Scores range from 57 to 113 in the ELEKT-D: Electroconvulsive Therapy (ECT) vs Ketamine in Patients With Treatment Resistant Depression (TRD) (ELEKT-D) trial, with higher scores indicating higher premorbid intelligence.

^d^ Significant after false discovery rate correction for multiple comparisons.

^e^ Scores range from 0 to 30, with higher scores indicating normal cognition.

^f^ Scores range from −11 to 62 in ELEKT-D, with higher scores indicating better functioning.

^g^ A 10-item clinician-rated scale, ranging from 0 to 60, in which a score greater than 36 indicates very severe depression.

While not significant after FDR adjustment, the baseline estimate of premorbid intelligence from the NAART-35 score was associated with differential rates of response with ketamine vs ECT based on the QIDS-SR16 score (χ^2^ = 5.92; unadjusted *P* = .02) and the MADRS score (χ^2^ = 4.61; unadjusted *P* = .03), as well as remission based on the QIDS-SR16 score (χ^2^ = 6.17; unadjusted *P* = .01) at the end-of-treatment visit. Among individuals with an NAART-35 score of less than 85, rates of response with ketamine (42.7% based on the QIDS-SR16 and 42.3% based on the MADRS) were higher than those with ECT (20.3% based on the QIDS-SR16 and 20.4% based on the MADRS). Similarly, rates of remission with ketamine (29.4% based on the QIDS-SR16 and 29.8% based on the MADRS) were higher than those with ECT (9.2% based on the QIDS-SR16 and 12.6% based on the MADRS). While ketamine always had numerically higher response and remission rates compared with ECT, the difference between these 2 treatment groups was lower among those with an NAART-35 score of 85 or more compared with those with a score of less than 85 (also see eFigure 3 in Supplement 2). Concurrent use of an atypical antipsychotic medication was associated with differential rates of remission based on the MADRS (χ^2^ = 5.50; unadjusted *P* = .02) with ketamine vs ECT; among those receiving concurrent atypical antipsychotic medication treatment, remission rates were 42.9% with ketamine and 10.6% with ECT (see eTable 2 in Supplement 2 for the results of analyses of associations with differential rates of response and remission with ketamine vs ECT based on both the QIDS-SR16 and the MADRS). Furthermore, the baseline MADRS was associated with differential improvement in the MADRS with ketamine vs ECT. Participants with a MADRS score of 36 or less at baseline had greater reduction with ketamine vs ECT (see eFigure 4 in Supplement 2). Conversely, those with very severe depression (ie, a MADRS score of more than 36^25^) at baseline had greater reduction in the MADRS score by week 2 with ECT vs ketamine, but the 2 groups were similar at the end-of-treatment visit.

### Baseline Features Associated With Improvement With ECT

**Figure 2.**
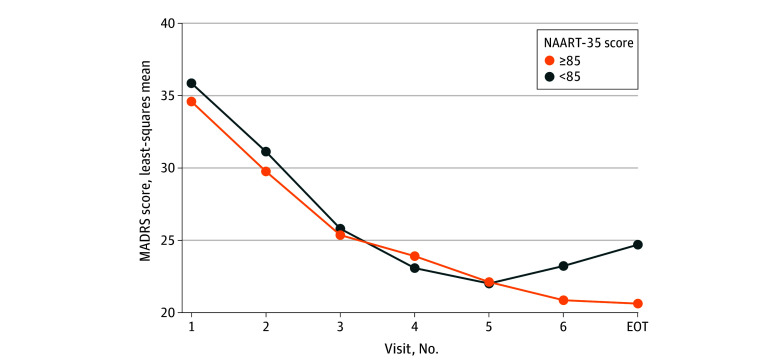
Differential Improvement in Clinician-Rated Depression Severity With Electroconvulsive Therapy (ECT) Based on the North American Adult Reading Test-35 (NAART-35), a Premorbid Intelligence Measure Clinician-rated depression severity was measured with the Montgomery-Åsberg Depression Rating Scale (MADRS), a 10-item scale, in which scores range from 0 to 60, with more than 36 indicating very severe depression. The binary outcome for NAART-35 was assigned using a standard score of less than 85 (low average or below), indicating scores that were 1.5 SDs or more below published normative data by the developers of NAART-35, in which scores range from 57 to 113 in the ELEKT-D: Electroconvulsive Therapy (ECT) vs Ketamine in Patients With Treatment Resistant Depression (TRD) trial, with higher scores indicating higher premorbid intelligence. EOT indicates end-of-treatment visit.

After FDR adjustment, there were significant baseline feature-by-time interactions in mixed-effects model analyses with the MADRS as the dependent variable only for the NAART-35, a comorbid PTSD diagnosis, and the HVLT-R delayed recall T score (see Table 3 for details). There was greater reduction in the MADRS score with ECT among individuals with a higher NAART-35 score (ie, scores ≥85) at the end-of-treatment visit compared with those with lower NAART-35 scores (ie, scores <85) (see Figure 2). Patients with comorbid PTSD experienced greater improvement in depression severity with ECT compared with those without comorbid PTSD (see eFigure 5 in Supplement 2). Among patients with impaired recall (ie, a lower HVLT-R delayed recall T score), there was greater reduction in depression severity during the second week of treatment, but the levels were similar to those with unimpaired recall at the end-of-treatment visit (see eFigure 6 in Supplement 2).

**Table 3.** Associations of Baseline Features With Changes in Depression Severity in Separate Analyses for ECT and Ketamine Treatment Groups

Treatment-by-time interaction	QIDS-SR16 as the measure^a^			MADRS as the measure^b^		
	*F* test	*df* ^c^	*P* value	*F* test	*df* ^c^	*P* value
**ECT**						
NAART-35 standard score^d^	1.79	6/901	.10	3.63	6/901	.001^e^
MoCA score^f^	0.62	6/913	.72	1.79	6/911	.10
HVLT-R delayed recall T score^g^	2.02	6/913	.06	3.94	6/911	<.001^e^
Concurrent benzodiazepine use	1.25	6/913	.28	0.87	6/912	.52
Concurrent atypical antipsychotic medication use	0.90	6/913	.50	0.94	6/912	.47
BMI	2.62	6/868	.02	0.28	6/867	.95
History of suicide attempt	2.28	6/913	.03	1.30	6/911	.26
Presence of anxious features	0.94	6/912	.47	0.70	6/911	.65
Comorbid PTSD diagnosis	1.34	6/912	.24	3.50	6/911	.002^e^
**Ketamine **						
NAART-35 standard score^d^	1.21	6/1103	.30	0.57	6/1104	.76
MoCA score^f^	2.08	6/1108	.05	2.17	6/1110	.04
HVLT-R delayed recall T score^g^	1.34	6/1103	.24	0.54	6/1104	.78
Concurrent benzodiazepine use	0.41	6/1108	.88	1.18	6/1110	.32
Concurrent atypical antipsychotic medication use	0.67	6/1108	.68	0.90	6/1110	.50
BMI	2.95	6/1072	.01	2.88	6/1074	.009
History of suicide attempt	1.29	6/1108	.26	0.75	6/1109	.61
Presence of anxious features	0.20	6/1108	.98	1.86	6/1110	.09
Comorbid PTSD diagnosis	1.13	6/1108	.34	1.18	6/1110	.31

Abbreviations: BMI (calculated as weight in kilograms divided by height in meters squared); ECT, electroconvulsive therapy; HVLT-R, Hopkins Verbal Learning Test-Revised; MADRS, Montgomery-Åsberg Depression Rating Scale; MoCA, Montreal Cognitive Assessment; NAART-35, North American Adult Reading Test-35; PTSD, posttraumatic stress disorder; QIDS-SR16, 16-item Quick Inventory of Depressive Symptomatology Self-Report.

^a^ Scores range from 0 to 27, with more than 20 indicating very severe depression.

^b^ A 10-item clinician-rated scale, ranging from 0 to 60, in which a score greater than 36 indicates very severe depression.

^c^ Presented as numerator *df*/denominator *df*.

^d^ Scores range from 57 to 113 in the ELEKT-D: Electroconvulsive Therapy (ECT) vs Ketamine in Patients With Treatment Resistant Depression (TRD) (ELEKT-D) trial, with higher scores indicating higher premorbid intelligence.

^e^ Significant after false discovery rate correction for multiple comparisons.

^f^ Scores range from 0 to 30, with higher scores indicating normal cognition.

^g^ Scores range from −11 to 62 in ELEKT-D, with higher scores indicating better functioning.

In results that were not adjusted for multiple comparisons, the presence of anxious features was associated with lower likelihood (odds ratio [OR], 0.41; 95% CI, 0.19-0.85) of response based on the MADRS, whereas initiation of treatment as inpatient was associated with higher likelihood (OR, 3.67; 95% CI, 1.28-10.49) of response based on the QIDS-SR16. Furthermore, higher baseline QIDS-SR16 levels were associated with lower likelihood of remission based on the QIDS-SR16 (OR, 0.62; 95% CI, 0.41-0.93) (see eTable 3 in Supplement 2 for results of logistic regression analyses identifying associations with response and remission at the end-of-treatment visit). In mixed-effects model analyses, a higher BMI was associated with greater reduction in the QIDS-SR16 with ECT (see eFigure 7 in Supplement 2).

### Baseline Features Associated With Improvement With Ketamine

There were no significant associations of changes in depression severity with ketamine after FDR adjustment. In unadjusted analyses, a higher QIDS-SR16 score was associated with lower likelihood of remission (OR, 0.49; 95% CI, 0.35-0.70), whereas a higher BMI was associated with higher likelihood of remission (for 1 SD difference: OR, 1.63; 95% CI, 1.15-2.30) (also see eTable 3 in Supplement 2). In mixed-effects model analyses, a higher BMI was associated with greater reduction in both the QIDS-SR16 and the MADRS with ketamine (also see eFigure 7 in Supplement 2). Additionally, a lower MoCA score at baseline was associated with greater decline of the MADRS in the ketamine treatment group.

## Discussion

This study, a post hoc secondary analysis of the ELEKT-D randomized clinical trial, aimed to answer 2 pivotal questions: (1) Can baseline clinical features identify individuals who experience greater improvement with ketamine vs ECT? (2) Within each treatment group, are baseline clinical features associated with acute-phase treatment outcomes? Our study found that ketamine was associated with greater treatment response than ECT among those with a QIDS-SR16 score of 20 or less (ie, moderately severe or severe) and those initiating treatment as outpatients. Within the ECT treatment group, higher estimates of premorbid intelligence and the presence of comorbid PTSD were associated with greater reduction in the MADRS. Those with lower T scores on the HVLT-R delayed recall had a greater reduction in clinician-rated depression severity during the second week of treatment, but the levels of MADRS were similar to those with unimpaired recall at the end-of-treatment visit. No other analyses were significant after controlling for multiple comparisons.

Findings of this study are consistent with the existing literature. Specifically, initiation of treatment in the inpatient setting was associated with better outcomes with ECT, which is consistent with previous findings.^26^ Rates of improvement with ECT were lower among those with lower scores on the NAART-35, which is consistent with prior studies in which lower educational levels were associated with poorer outcomes with ECT.^19^ Our finding that a higher BMI was associated with better outcomes with ketamine (the average dose of ketamine at each visit of ELEKT-D was 0.5 mg/kg^10^) is consistent with a recent meta-analysis of pooled studies from single-infusion ketamine studies,^24^ but the present study is the first, to our knowledge, within the context of an acute course (6 infusions over 3 weeks). These findings add to the growing literature on the potential association between obesity and response to antidepressant treatments.^27,28,29,30,31^ However, unlike previous studies, there was no association between concurrent benzodiazepine use and outcomes with ketamine.^17,21,22,23^ A potential reason could be that dose-related effects of concurrent benzodiazepines were not evaluated in the present study.

Findings of this study may inform shared decision-making approaches for patients with TRD and their clinicians. While the primary study of ELEKT-D demonstrated noninferiority of ketamine compared with ECT, this study suggests that ketamine may be especially preferred over ECT among those with TRD who have moderately severe or severe depression or who are initiating treatment as outpatients. Furthermore, use of an estimate of premorbid intelligence (such as the NAART-35) may be informative. Among individuals with scores on this test that are 1 SD or more below the normal score, 1 additional response may be achieved by treating 4 or 5 additional patients with ketamine vs ECT, and 1 additional remission may be achieved by treating 5 or 6 additional patients with ketamine vs ECT. These findings regarding differential benefits of ketamine vs ECT along with the considerations regarding risks and burden associated with ketamine and ECT should be incorporated in shared decision-making approaches for TRD.

### Limitations

This study has several limitations. The ELEKT-D trial was not designed to detect differences in outcomes between ECT and ketamine based on these baseline features, so these analyses may not have been adequately powered. As these were post hoc analyses, the findings should be considered preliminary and warrant replication before larger-scale clinical implementation. This study was focused on a limited set of features that were informed by existing literature and potentially missed out on other features, such as anxiety, rumination, inattention, and borderline personality diagnosis or traits, that could have been associated with differential treatment outcomes. Furthermore, use of precision psychiatry approaches using biomarkers such as those of neural circuit dysfunction^32^ may further inform treatment selection of ketamine vs ECT at an individual level. The findings of the ELEKT-D trial may have been limited by low enrollment of patients who were responsive to ECT (such as inpatients, older patients, and patients who are depressed with psychosis)^33^ and by the relatively short course length for ECT compared with the common clinical practice. Given the higher dropout rate after randomization to ECT (33 of 203 participants) vs ketamine (5 of 200 participants), generalizability may be limited given that identifying an association with a treatment response would have been unavailable for those who did not complete any posttreatment assessments. An additional limitation of the ELEKT-D trial is that it did not collect biological markers that may have been relevant, as prior research suggests that brain- and blood-based biomarkers may have utility in guiding selection among commonly used antidepressants.

## Conclusions

In this secondary analysis of the ELEKT-D randomized clinical trial of ECT vs ketamine, greater reductions in depression severity were observed with ketamine among outpatients as well as those with moderately severe or severe depression severity. Therefore, shared decision-making approaches for selecting between ECT and ketamine may incorporate findings from this study. Future studies are needed to replicate and extend these findings to inform selection of optimal therapy by patients with TRD and their clinicians.

## Notes

Supplement 1.
Trial Protocol

Supplement 2. **eFigure 1.** CONSORT Diagram of ELEKT-D Participants Included in This Report **eFigure 2.** Changes in QIDS-SR16 With Ketamine vs ECT Based on Hospital Admission Status at the Time of First Treatment **eFigure 3.** Differential Rates of Response and Remission With Ketamine vs ECT Based on Standardized Scores on the North American Adult Reading Test 35 (NAART-35) **eFigure 4.** Treatment Effects of Ketamine vs ECT Based on MADRS Stratified by Less (Left Panel) or More (Right Panel) Severe Baseline Depression Severity **eFigure 5.** Treatment Effects of ECT Based on Presence of Comorbid PTSD **eFigure 6.** Treatment Effects of ECT Based on HVLT-R Levels at Baseline **eFigure 7.** Treatment Effects of Ketamine and ECT Based on Body Mass Index (BMI) Categories **eTable 1.** Baseline Feature of ELEKT-D Participants Randomized to ECT Who Dropped Out (ie, Did Not Complete Any Posttreatment Assessment) vs Those Who Did Not **eTable 2.** Baseline Features That Predict Response and Remission With ECT vs Ketamine **eTable 3.** Baseline Features That Predict Response and Remission in Separate Analyses for ECT and Ketamine Treatment Groups

Supplement 3.
Data Sharing Statement
