# Supplementary material for: Ketamine vs Electroconvulsive Therapy for Treatment-Resistant Depression: A Secondary Analysis of a Randomized Clinical Trial
Source: JAMA Netw Open. 2024 Jun 25;7(6):e2417786. doi: 10.1001/jamanetworkopen.2024.17786 (PMC11200139; doi:10.1001/jamanetworkopen.2024.17786)
Supplement: Supplement 1. — Trial Protocol [file jamanetwopen-e2417786-s001.pdf]

ELEKT-D: Electroconvulsive therapy (ECT) vs. ketamine in patients with treatment resistant depression (TRD)

Version Date: 02FEB2017
Protocol Version: 3.0
Sponsor: Cleveland Clinic Foundation
Funding Agency: PCORI (Patient Centered Outcomes Research Institute)
Principal Investigator: Amit Anand, MD

This investigational protocol contains confidential information for use by the principal investigators and their designated representatives participating in this clinical investigation. It should be held confidential and maintained in a secure location. It should not be copied or made available for review by any unauthorized person or firm.

Signature Page

Study Title:

ELEKT D: Electroconvulsive therapy (ECT) vs. ketamine in patients with treatment resistant depression (TRD)

Version date: 02FEB2017

Protocol Version: 3.0

I, the undersigned, have read and approve this protocol and agree on its contents. It is confirmed that the information and guidance given in this protocol complies with scientific principles, the guidelines of Good Clinical Practice, the Declaration of Helsinki in the latest relevant version, and the applicable legal and regulatory requirements.

Principal Investigator
Signature

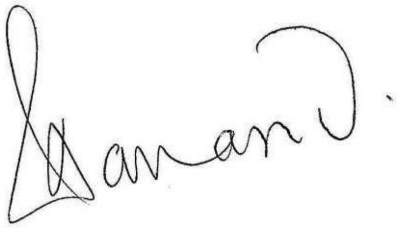

Amit Anand, MD

02FEB2017

Date

Table of Contents

1 Table of Contents

1. Introduction **11**

2. Background and Rationale **11**

3. Study Design **12**

4. Outcome Measures **13**

4.1 Primary Outcome *13*

4.2 Secondary Outcomes *14*

5. Subject Selection **14**

5.1 Recruitment of Trial Participants *14*

5.2 Inclusion Criteria *14*

5.3 Exclusion Criteria *15*

5.4 Randomization of Patients *15*

6. Study Treatments **16**

6.1 Acute Treatment Phase (To occur over 3 to 5 weeks) *16*
6.1.1 ECT Arm 16
6.1.2 Ketamine Arm 16

6.2 Assessment for Response during Acute Treatment Phase *17*
6.2.1 Responders 17
6.2.2 Non Responders 17

6.3 Concomitant Medication *17*
6.3.1 Medications Allowed During Study 17
6.3.2 Prohibited Medications 17

7. Study Procedures **18**

7.1 Screening Visit (To occur within 28 days of referral for clinical ECT) *18*
7.1.1 Screen Failures 19

7.2 Baseline Visit/Visit 1 (To occur within 1 week after the Screening Visit) *19*

7.3 Treatment Visits (To occur over 3 to 5 weeks) *20*
7.3.1 ECT Arm 20
7.3.2 Ketamine Arm 20
7.3.3 Completion and/or Early Termination of Treatment Phase 21

7.4 End of Treatment (EOT) Visit *21*

7.5 Follow Up Visits for Responders *23*
7.5.1 Month 1 Follow Up Visit (+/ 2 weeks) 23
7.5.2 Month 3 Follow Up Visit (+/ 2 weeks) 23
7.5.3 Month 6 Follow Up Visit (+/ 2 weeks) 24

7.6 Non Compliance *25*
7.7 Subject Withdrawal *25*
7.8 Lost to Follow Up *25*
7.9 Study Termination *26*
8. Safety Monitoring and Reporting **26**
8.1 Unexpected Adverse Events *26*
8.2 Study Treatment Discontinuation Adverse Events *27*
8.3 Serious Adverse Events *27*
8.4 Documentation and Reporting of Serious Adverse Events *27*
9. Statistical Plan **28**
10. Study Committees **28**
11. Data Handling and Record Keeping **29**
11.1 Data Collection *29*
11.2 Retention of Records *29*
12. Study Monitoring, Auditing, and Inspecting **30**
Study Monitoring Plan *30*
13. Ethical Considerations **30**
14. Publication and Disclosure **31**
15. References **32**
16. Appendix **33**
List of Questionnaires Schedules of Events

List of Abbreviations

AE	Adverse Event
ATHF	Antidepressant Treatment History Form
BCM	Baylor College of Medicine
BPRS	Brief Psychiatric Rating Scale
GCI S/CGI I	Clinical Global Impression Scale for Severity and Improvement
CCBH	Cleveland Clinic Center for Behavioral Health
CNRU	Clinical Neuroscience Research Unit (Yale)
COWAT	Controlled Oral Word Association Test
CSSRS	Columbia Suicide Severity Rating Scale
C5R	C5Research (Cleveland Clinic)
CADSS	Clinician Administered Dissociative Symptoms Scale
CPFQ	Cognitive and Physical Functioning Questionnaire
DSMB	Data Safety Monitoring Board
DSM 5	Diagnostic and Statistical Manual of Mental Disorders (5th Ed.)
ECT	Electroconvulsive Therapy
eCRF	Electronic Case Report Form
EOT	End of Treatment
EC	Executive Committee
GSE My	Global Self Evaluation of Memory
HVLT R	Hopkins Verbal Learning Test
IRB	Institutional Review Board
ITT	Intent to Treat
MADRS	Montgomery Asberg Depression Rating Scale
MAOI	Monoamine Oxidase Inhibitor
MAP	Mt. Sinai Mood and Anxiety Disorders Program
MINI 7.0	Mini Neuropsychiatric Interview
MoCA	Montreal Cognitive Assessment
MDD	Major Depressive Disorder
MSSM	Mount Sinai Medical Center
NAART	North American Adult Reading Test
PGIC/PGII	Patient Global Impression Scale
PRISE	Patient Rated Inventory of Side Effects
QIDS SR 16	Quick Inventory of Depressive Symptoms
QOLS	Quality of Life Scale
SAE	Serious Adverse Event
SAP	Statistical Analysis Plan
SC	Stakeholder Committee

122

SOC	Standard of Care
SMCQ	Squire Memory Complaint Questionnaire
SSRI	Selective Serotonin Reuptake Inhibitor
TRD	Treatment Resistant Depression
YMRS	Young Mania Rating Scale

Study Synopsis

Title	ELEKT D: Electroconvulsive therapy (ECT) vs. ketamine in patients with treatment resistant depression (TRD)
Sponsor	Cleveland Clinic Foundation
Funding Source	PCORI (Patient Centered Outcomes Research Institute)
Study Centers	There will be approximately four study sites located throughout the U.S.
Patient Population	Outpatients or inpatients with non psychotic TRD referred by their clinical providers and eligible for ECT treatment.
Study Objectives	The aim of the study is to conduct a comparative randomized trial of ECT versus ketamine for TRD in a real world setting with patient reported outcomes as primary and secondary outcome measures.<br><br>**Specific Aim 1:** To investigate the comparative effectiveness of ECT and ketamine on measures of depression.<br>**Specific Aim 2:** To investigate the relative impact of ECT and ketamine on measures of memory and cognitive function.<br>**Specific Aim 3:** To investigate the relative impact of ECT and ketamine on patient reported quality of life measures after acute treatment and at follow up over six months.
Study Design	This is an unblinded prospective randomized open label clinical trial. Patients will be randomized 1:1 to receive either ECT 3 times per week or ketamine 2 times per week over 3 weeks (additional 2 week window allowed for flexibility).<br><br>Responders (patients who achieve a 50% decrease on the QIDS SR 16 score from Baseline to the End of Treatment visit) will return for three follow up visits over a six month period following the end of treatment visit. Non responders will not be followed after the End of Treatment visit.
Number of Patients	Approximately 400 eligible subjects will be enrolled, 200 in the ECT arm and 200 in the ketamine arm.<br><br>It is estimated that 60% or 240 patients (approximately 120 in each arm) will be classified as responders. Due to the expected high level of attrition, 192 patients classified as responders (approximately 96 in each arm) are expected to complete the follow up visits.
Duration of patient	The screening period will be a maximum of 28 days. Randomization will occur within 1 week after the screening period after eligibility is confirmed.

participation and duration of the study	After patients are enrolled they will be randomized to either ECT or ketamine therapy. Patients in the ECT arm will receive up to 9 treatments over 3 weeks (+ 2 weeks). Patients in the ketamine arm will receive up to 6 treatments over 3 weeks (+ 2 weeks).<br><br>Patients in both arms classified as responders will have 3 additional visits, at 1 month, 3 months, and 6 months after the End of Treatment visit. Responders are patients who achieve a 50% decrease in QIDS SR score from Baseline to the End of Treatment visit.
Key Selection Criteria	**INCLUSION CRITERIA**<br>1. Written informed consent before any study related procedures are performed<br>2. Inpatients or outpatients referred by their providers for ECT treatment and eligible for ECT treatment<br>3. Males/females at least 21 years of age but no older than 75 years of age<br>4. Meet DSM 5 criteria for Major Depressive Episode in a as determined by both:<br>A. clinician's diagnostic evaluation and<br>B. confirmed with the MINI International Neuropsychiatric Interview (MINI 7.0)<br>5. A current depressive episode that has lasted a minimum of 4 weeks<br>6. Meet all of the following criteria on symptom rating scales at screening:<br>A. Montgomery Asberg Depression Rating Scale (MADRS) score >20<br>B. Young Mania Rating Scale (YMRS) of 5<br>C. Montreal Cognitive Assessment (MoCA) of 18<br>7. Have had 2 adequate trials of antidepressants or augmentation strategies during their lifetime (Refer to ATHF Guidelines for Completion for guidelines on dose/duration required for a trial to be considered adequate.)<br>8. In the opinion of the investigator, the patient is willing and able to comply with scheduled visits, treatment plan, and other trial procedures for the duration of the study<br><br>**EXCLUSION CRITERIA**<br>1. Meet DSM 5 criteria for bipolar disorder, schizophrenia, schizophreniform disorder, schizoaffectivedisorder, mental retardation, or pervasive developmental disorder<br>2. Meets any exclusion criteria for ECT or ketamine treatment as described in the clinical guidelines or according to investigator judgment

	3. The patient is pregnant or breast feeding<br>4. The patient has a severe medical illness or severe neurological disorder<br>5. The patient has a known ketamine allergy or is taking a medication that may interact with ketamine<br>6. Diagnosis of major depressive disorder with psychotic features during the current depressive episode<br>7. Unable to give informed consent<br>8. Was previously enrolled/randomized into the trial
Test Product, Dose, and Mode of Administration	ECT:<br>Patients randomized to the ECT arm will receive standardized ECT treatment as determined by each study site. The starting ECT treatment will be Right Unilateral (RUL) ultra brief pulse at 6X seizure threshold determined during titration at the first visit. After RUL for four to six treatments, if there is not satisfactory improvement, there will be a switch to Bilateral (BL) utilizing brief pulse using 0.5 modified half age method to determine stimulus intensity.<br>Anesthesia will be administered according to standard of care at each site, but ketamine will not be allowed. Patients will receive up to nine treatments over 3 weeks (additional 2 week window allowed for flexibility).<br><br>Flexibility will be allowed for the clinician to adjust the treatment as clinically necessary.<br><br>Ketamine:<br>The standard dose of ketamine (0.5mg/kg infusion over 40 minute period) will be administered 2 times per week over 3 weeks (additional 2 week window allowed for flexibility). The investigator will be able to adjust the dose if clinically warranted.<br><br>Flexibility will be allowed for the clinician to adjust the treatment as clinically necessary.
Concomitant Medications	All patients will continue their existing antidepressant treatment while on the study protocol. Patients will also continue existing non psychotropic medications initiated prior to the baseline visit, unless the investigator determines that they are contraindicated for ECT or ketamine treatment. Each site will follow their standard clinical protocol for this.<br><br>Investigators will follow their site�s standard safety evaluation process for anesthesia administration and ECT.
Prohibited Medications	Any medication that is judged by the investigator to have significant clinical interaction with ECT or ketamine.

Outcome Measures	The primary outcome measure is response rate, defined as 50% reduction in QIDS SR16 scores from Baseline Visit to the End of Treatment Visit.
Secondary Outcome Measures	Secondary outcome measures will include clinician and patient rated scales for depression, suicidality, cognition, and associated psychiatric symptoms.
AE/ SAE Collection	**SAEs will be reported to the sponsor and the respective site IRB within 24 hours of notification of the event.**<br><br>SAEs<br>Death<br>Life threatening AEs (including suicide attempt)<br>A new inpatient hospitalization or prolongation of existing hospitalization.<br>A disability/incapacity<br>A congenital anomaly/birth defect in the offspring of a patient who received drug<br>Other Serious Event (Important Medical Event) an important medical event that may jeopardize the subject or may require medical intervention to prevent one of the outcomes listed above. Medical and scientific judgment should be exercised in deciding<br>whether other situations should be considered serious adverse event.
Statistical Methodology	The primary analyses will be performed on the modified intention to treat (mITT) population defined as a randomized patient having at least one treatment and one QIDS SR16 measurement during the acute treatment phase. The primary outcome measure of response rate will be compared between ketamine and ECT using a chi square test, to test the comparative efficacy of ketamine and ECT for reduction in depressive symptoms. A multivariable logistic regression model will be constructed for the analysis, to account for potential heterogeneity of treatment effect caused by confounding variables. A similar analytic strategy will be applied to evaluate cognitive function and quality of life.

1. Introduction

Major depressive disorder (MDD) accounts for 65.5 million disability adjusted life years (DALYs) and ranks third among illnesses of global disease burden (1). Identifying treatments that are more effective for MDD is required to meet this large and growing public health challenge. However, recent data suggest that antidepressant therapies are less efficacious than previously thought (2, 3). The real world effectiveness of antidepressants is sub optimal in approximately two of three patients (4, 5). TRD has been defined as depression resistant to one or more adequate trials of antidepressants for which the patient reports minimal or no significant improvement in mood (6, 7). TRD has been noted to be present in 20 50% of depression patients. TRD patients have significantly higher outpatient costs and a six higher cost of hospitalization (8). Chronic, inadequately treated depression is associated with loss of social and workplace functioning, increased medical illnesses and healthcare use, and an increased risk for suicide(6). In the United States, the total economic cost of depression in 2012 was estimated at $188 billion (6). Hence, there is an urgent need to identify treatments that can be effective for TRD.

2. Background and Rationale

<u>ECT Use in TRD</u>

ECT has been in use for nearly 75 years for severe TRD and is considered to be one of the most effective treatments (9). However it is associated with a number of side effects and social stigma.

Cognitive impairment and significant memory loss has been observed in unilateral and bilateral as well as higher dose ECT immediately after treatment (10). Additional adverse events associated with ECT include anterograde amnesia (i.e. memory disturbance of events after ECT treatment) in the short term and retrograde amnesia (memory disturbance of events before ECT treatment) in the long term. Retrograde amnesia can persist for years after ECT treatment particularly for events near the time of treatment (11).

Other side effects of ECT include risks of receiving general anesthesia and muscle relaxants, delirium in the post ECT period, headaches and muscle aches, nausea and fatigue and rarely, prolonged seizures.

<u>Ketamine as an Alternative Treatment for TRD</u>

Ketamine shows promise as a treatment for TRD, but there is an evidence gap in its use as an alternative to ECT. Ketamine is a sedative/analgesic and general anesthetic approved by the FDA for human and veterinary use. It is an antagonist of the N methyl d aspartate (NMDA) receptors in the brain and decreases the neurotransmission of glutamate (the main excitatory neurotransmitter) via the NMDA receptor.

Single infusion of a subanesthetic dose of ketamine has shown rapid but transient reversal of TRD symptoms. A number of open label studies done so far indicate that repeated ketamine infusion treatment results in responses similar to that of ECT (40). Ketamine can lead to symptoms of dissociation, perceptual disturbances, or even psychotic like symptoms (33) although these are seen infrequently at subanesthetic doses used in the treatment of TRD and are rapidly reversed after stopping the infusion.

Currently, there are no formal randomized trials with detailed clinical and demographic data to provide direct comparative efficacy evidence between ECT and ketamine treatment. This study will provide such data and help to fill the evidence gap for efficacy of ECT and ketamine treatment for TRD.

3. Study Design

This is a prospective randomized open label 2 arm (1:1) clinical trial of TRD with either ECT or ketamine treatment. Given the nature of ketamine and ECT treatments randomization and treatment arms cannot be blinded at the clinician or patient level.

After screening and evaluation of inclusion/exclusion criteria, patients will be randomized to either ECT three times per week or ketamine two times per week. This acute treatment phase will last between three to five weeks. This timeframe allows for changes in the treatment schedule due to clinician discretion or the patient's schedule. Patients may respond or remit to ECT or ketamine before they have completed all treatment visits and therefore may not undergo the full nine visits (for ECT) or six visits (for ketamine). Investigators will closely monitor patients and may adjust treatments during the acute treatment phase at any time. All patients, regardless of how many treatment visits have been completed, should complete an End of Treatment Visit.

All patients will complete self reported cognitive assessments, depression questionnaires, and quality of life scales at the Baseline Visit and throughout the acute treatment phase of the study (see Schedule of Events). Diagnostic interviews and clinician rated scales will also be performed at regular intervals throughout the acute treatment phase.

After the acute treatment phase, all patients will complete an end of treatment (EOT) visit within one week of their last study treatment and will be classified as either a responder or a non responder. Patients may continue to receive ECT or ketamine clinically after the EOT visit, but this will mark the end of the acute treatment phase of the study. If the patient is a responder any ECT or ketamine treatments administered after the EOT visit will be recorded in the follow up phase. If the patient is not a responder this will conclude their participation in the study and they will be treated clinically.

- Responder: a patient who achieves a ≥ 50% decrease in their QIDS SR 16 score from Baseline to the End of Treatment visit. Responders will continue in the study for three follow up visits at one month, three months, and six

months after the EOT Visit. They will continue naturalistic treatment with a clinician of their choice.

- Non responder: a patient who achieves <50% decrease in their QIDS SR 16 score from Baseline to the End of Treatment Visit. Non responders will be exited from the study after the End of Treatment Visit and will not be seen for follow up visits. They will continue treatment with the clinician of their choice

Early Completion of Acute Treatment Phase

The investigator or patient can choose to stop treatment at any time. Investigators will monitor patients closely for signs of improvement, remission, or decline. The investigator will use his or her discretion and clinical judgment to determine if a patient should stop treatment and be scheduled for an End of Treatment Visit.

Investigators may decide to stop treatment for patients who show improvement after less than nine ECT treatments or six ketamine treatments. These patients should be scheduled for an End of Treatment Visit and if they are found to be responders will participate in the follow up visits.

Investigators may also decide to stop treatment for patients who decline or have worsening depression or suicidality during the acute treatment phase. These patients should be scheduled for an End of Treatment Visit and be evaluated for response. (These patients are unlikely to be classified as responders.)

If an outpatient has worsening depression that requires psychiatric hospitalization, the patient may continue in the study at investigator discretion. These patients can receive study ECT or ketamine treatments as an inpatient.

4. Outcome Measures

To avoid potential bias, the patient assessments and the clinician assessments should be completed independently and without reference to one another. The research coordinator or clinician administering the questionnaires should not view the patient's responses on patient rated scales. (They should, however, remind the patient to answer all questions and not leave any questions blank.)

4.1 Primary Outcome

The primary outcome measure is the percent of responders. Treatment response is defined as a ≥ 50% decrease in QIDS SR 16 scores from the Baseline Visit to the EOT visit

The QIDS SR 16 will be administered prior to treatment according to the following schedules. ECT Arm

QIDS SR 16 will be administered at certain time points during the acute treatment phase (Baseline/Visit 1, Visit 2, Visit 4, Visit 6, Visit 7, and Visit 9, EOT visit) Patients in the ECT arm who are classified as responders will complete the QIDS SR 16 at all follow up visits.

<u>Ketamine Arm</u>

The QIDS SR 16 will be administered at all visits during the acute treatment phase (Baseline/Visit 1, Visit 2, Visit 3, Visit 4, Visit 5, Visit 6, EOT visit). Patients in the ketamine arm who are classified as responders will complete the QIDS SR 16 at all follow up visits.

This scale should be the first assessment administered and should be checked for completeness by the study nurse or research coordinator. All attempts should be made to have a consistent and neutral atmosphere for the patient to complete the QIDS and all patient rated scales to minimize outside influence.

4.2 Secondary Outcomes

Other patient and clinician rated scales will be used as secondary outcome measurements. (See Table 1.)

All scales (except CADSS and BPRS) will be administered prior to treatment. CADSS and BPRS will be administered by the research nurse or a clinician post treatment. Questionnaires will be administered according to the schedule of events.

During the follow up visits, data analysis for time points 1, 3, and 6 months will be conducted using the End of Treatment Visit as baseline.

5. Subject Selection

5.1 Recruitment of Trial Participants

Inpatients or Outpatients with non psychotic TRD referred by their clinical providers for ECT and found to be eligible for ECT treatment will be pre screened for the study. Potential patients will be approached after a psychiatrist has evaluated them and recommended them for clinical ECT treatment. At this time the patient will be informed about the study and given a thorough explanation of risks, benefits, study procedures, and expectations.

Patients interested in participation will be scheduled for a screening visit within 2 weeks (but ideally as soon as possible)

5.2 Inclusion Criteria

Patients are eligible for the study if they meet the following inclusion criteria:

a) Written informed consent before any study related procedures are performed

b) Inpatients or outpatients referred by their providers for ECT treatment and eligible for ECT treatment
c) Males or females at least 21 years of age, but no older than 75 years of age
d) Meet DSM 5 criteria for a Major Depressive Episode as determined by both:
   A. a clinician's diagnostic evaluation and
   B. confirmed by interview using the Mini International Neuropsychiatric Interview (MINI)
e) A current depressive episode that has lasted a minimum of 4 weeks
f) Meet all of the following criteria on symptom rating scales at screening
   A. Montgomery Asberg Depression Rating Scale (MADRS) score > 20
   B. Young Mania Rating Scale (YMRS) ≤ 5
   C. Montreal Cognitive Assessment (MoCA) of ≥ 18
g) Have had ≥ 2 adequate trials of antidepressants/augmentation strategies during their lifetime. (Refer to ATHF Guidelines for Completion for guidelines on dose/duration required for a trial to be considered adequate.)
h) In the opinion of the investigator, the patient is willing and able to comply with scheduled visits, treatment plan, and other trial procedures for the duration of the study

5.3 Exclusion Criteria

Patients must NOT meet any of the following exclusion criteria:

a) Meets DSM 5 criteria for bipolar disorder, schizophrenia, schizophreniform disorder, schizoaffective disorder, mental retardation, or pervasive development disorder
b) Meet any exclusion criteria for ECT or ketamine treatment as described in the clinical guidelines or according to investigator judgment
c) The patient is pregnant or breast feeding
d) The patient has a severe medical illness or severe neurological disorder
e) The patient has a known ketamine allergy or is taking any medication that may interact with ketamine
f) Diagnosis of major depressive disorder with psychotic features during the current depressive episode
g) Unable to give informed consent
h) Was previously enrolled/randomized into the trial.

5.4 Randomization of Patients

All patients who are eligible for the trial will be randomized in a 1:1 fashion to either ECT or ketamine treatment. Given the nature of these treatments, treatment arms cannot be blinded at the patient or clinician level. Randomization will be conducted centrally through a secure electronic data management system. Detailed instructions can be found in the Manual of Operations (MOP).

6. Study Treatments

6.1 Acute Treatment Phase (To occur over 3 to 5 weeks)

6.1.1 ECT Arm

Patients will undergo anesthesia evaluation according to each site's standard clinical procedure. Anesthesia will be administered according to standard of care at each site, but ketamine will not be allowed.

The initial ECT treatment will be Right Unilateral (RUL) ultra brief pulse at 6X seizure threshold determined during titration at first visit. If there is not satisfactory improvement with RUL the investigator may change to Bilateral (BL) utilizing brief pulse using 0.5 modified half age method to determine stimulus intensity. The seizure threshold may increase during the course of treatment and the dose of the electric stimulus may need to be increased incrementally (16). It is suggested to change to bilateral after three to five RUL treatments (17).

Treatments will be given three times a week and after 9 treatments the acute arm of the study would be complete. Flexibility will be allowed for the ECT clinician to adjust the treatments as clinically necessary.

Patients will receive up to nine treatments over three to five weeks. Ideally patients will receive treatments at regular intervals of three times per week for three weeks. The window allows for modifications based on clinician discretion and the patient's schedule.

Patients will be assessed by clinical providers prior to each visit to evaluate treatment response and appropriateness for continued treatment. Patients will be assessed for any adverse events and treated per investigator discretion.

Patients will receive both patient rated and clinician rated behavioral scales at Baseline/Visit 1, Visit 2, Visit 4, Visit 6, Visit 7, Visit 9, and EOT Visit.

6.1.2 Ketamine Arm

Patients will receive up to six treatments over three to five weeks. Ideally patients will receive treatments at regular intervals of two times per week for three weeks. The window allows for modifications based on clinician discretion and patient schedules.

Ketamine will be administered according to the standard dose of 0.5mg/kg infusion over a 40 min period).The investigator will be able to modify the dose if clinically warranted.
Treatments will be given two times a week for a maximum of six treatments after which the acute arm of the study will be complete.

Patients will be clinically assessed prior to each treatment to evaluate response and appropriateness for continued treatment. Patients will be assessed for any adverse events and treated per investigator discretion.

Patients will receive both patient rated and clinician rated behavioral scales at all treatment visits (Baseline/Visit 1, Visit 2, Visit 3, Visit 4, Visit 5, Visit 6, and EOT Visit).

6.2 Assessment for Response during Acute Treatment Phase

6.2.1 Responders

Patients who have a decrease of 50% or more on the QIDS SR 16 from Baseline Visit to the EOT Visit will be classified as a responder. These patients will be included in the six month follow up phase.

The clinician may decide to stop treatment before the maximum number of visits and schedule the patient for an EOT Visit.

6.2.2 Non Responders

Patients who have less than a 50% decrease in QIDS SR 16 score from the Baseline Visit to the EOT Visit will be classified as non responders. These patients will not be included in the follow up portion of the trial. Patients will be referred to their clinical provider for ongoing treatment of depression. Continuity of care between study staff and clinical providers will be carefully managed in order to provide optimal ongoing psychiatric care for the patient.

6.3 Concomitant Medication

6.3.1 Medications Allowed During Study

Patients will be allowed to continue their existing psychotropic medications. Changes in psychotropic medications throughout the trial are best avoided but are permitted according to investigator discretion. These medications must be recorded on the psychiatric concomitant medication log.

Subjects should continue medications for other conditions and be reminded to tell study staff of any changes to medical history or concomitant medications. This will be assessed at each visit prior to treatment. All subjects should receive optimal care for any side effects occurring during the study (nausea, headache, etc.) according to local standards of care or evidence based guidelines, and at the discretion of the investigator.

6.3.2 Prohibited Medications

Patients should not be enrolled in the study if they are taking any medication that is contraindicated for ECT or ketamine treatment.

Changes in medical history and concomitant medications will be assessed at each visit and patients may be withdrawn from the study at any point if they are taking prohibited medication.

7. Study Procedures

7.1 Screening Visit (To occur within 28 days of referral for clinical ECT)

- Informed Consent: An investigator and/or other delegated study team member will discuss all risks and benefits of participation and review the study visit schedule with the patient. The patient will sign the informed consent form prior to any study procedures being performed.
- Assessment of Inclusion/Exclusion Criteria
- Demographics
- Medical History, including psychiatric history and history of neurological conditions
- Psychiatric Medication Log (current medications)
- Clinical Psychiatric Evaluation
- Diagnostic evaluation by investigator
    - Meets DSM 5 criteria for MDD, but does not meet DSM 5 criteria for Schizophrenia, Schizophreniform disorder, schizoaffective disorder, mental retardation, Pervasive Development Disorder
    - No psychotic episodes during the current depressive episode
    - Verify current depressive episode has lasted at least 4 weeks
    - Verify that patient has had at least 2 adequate (Refer to ATHF Guidelines for Completion for guidelines on dose/duration required for a trial to be considered adequate.) trials of antidepressant therapy
- Urine pregnancy test for females of child bearing potential
- Diagnostic Interviews
    - MINI
    - ATHF
- Clinician Rated Scales
    - MADRS
    - YMRS
- Cognitive Assessments
    - MoCA

If patients meet eligibility criteria they will be randomized into either the ECT or ketamine arm via a secure electronic data management system. Randomization can be done by study staff after the screening visit. Depending on which arm the patient is randomized to, the study nurse will schedule them for their Baseline/Visit 1 according to the site's clinical or research schedule.

Study staff should schedule patients for their Baseline/Visit 1 within one week of the Screening Visit.

7.1.1 Screen Failures

If the patient fails to qualify for the study after signing the informed consent form, they will be considered a screen failure. Screen failures should be recorded on the electronic screening log along with the reason for disqualification. Patients who screen fail and are not randomized will be eligible to rescreen at a later date. Patients should only be enrolled into the study once, therefore patients who have been randomized will not be eligible for future screening.

7.2 Baseline Visit/Visit 1 (To occur within 1 week after the Screening Visit)

- Update Medical History
- Update Psychiatric Medication Log
- Clinical Psychiatric Evaluation
- Vitals (BP, height, weight)
- Evaluation for AEs and SAEs

Questionnaires to be Completed Prior to Treatment •

- Patient Rated Behavioral Scales
    - QIDS SR 16 (to be completed first)
    - SMCQ
    - PGIC C
    - QOLS
    - PRISE
    - CPFQ
- Clinician Rate Behavioral Scales
    - MADRS
    - CSSRS
    - YMRS
    - CGI S
- Cognitive Assessments
    - COWAT
    - HVLT R
    - Stroop
    - NAART

ECT Procedure or Ketamine Infusion Questionnaires to be

Completed Post Treatment*

- CADSS
- BPRS

*Patients in the ECT arm will need to have a recovery period prior to completing the post treatment questionnaires.

7.3 Treatment Visits (To occur over 3 to 5 weeks)

7.3.1 ECT Arm

Prior to each treatment, patients will be assessed if clinically appropriate for the treatment. Components of the evaluation for ECT will vary on a case by case basis. Each site will perform the following minimal set of assessments:

- Update Medical History
- Update Psychiatric Medication Log
- Vitals (BP, heart rate, weight)
- Evaluation for Adverse Events or Serious Adverse Events
- Clinical Psychiatric Evaluation
- Patient and Clinician Rated Behavioral Scales (to be completed at Baseline/Visit 1, Visit 2, Visit 4, Visit 6, Visit 7, and Visit 9.

Prior to Treatment:

- Patient Rated Behavioral Scales (to be completed at every visit)
  - QIDS SR 16 (to be completed first)
  - GSE MY
  - SMCQ
  - PGIC C & PGIC I
  - QOLS
  - PRISE
  - CPFQ

Clinician Rated Behavioral Scales

  - MADRS
  - CSSRS
  - YMRS
  - CGI S & CGI I

- Post Treatment (clinician rated)
  - CADSS
  - BPRS

7.3.2 Ketamine Arm

Prior to each treatment, patients will be assessed if clinically appropriate for the treatment. Components of the evaluation for Ketamine will vary on a case by case basis.
Each site will perform the following minimal set of assessments: •

Update Medical History

- Update Psychiatric Medication Log
- Vitals (BP, heart rate, weight)

- Evaluation for Adverse Events or Serious Adverse Events
- Clinical Psychiatric Evaluation
- Patient and Clinician Rated Behavioral Scales (to be completed at every visit)

Prior to Treatment:

- Patient Rated Behavioral Scales (to be completed at every visit)
  - QIDS SR 16 (to be completed first)
  - GSE MY (not completed at Baseline/Visit 1)
  - SMCQ
  - PGI C & PGI I (PGI I not completed at Baseline/Visit 1)
  - QOLS
  - PRISE
  - CPFQ
- Clinician Rated Behavioral Scales (to be completed at every visit)
  - MADRS
  - CSSRS
  - YMRS
  - CGI S & CGI I (CGI I not completed at Baseline/Visit 1)

Post Treatment (clinician rated):

  - CADSS
  - BPRS

7.3.3 Completion and/or Early Termination of Treatment Phase

Investigators can end the treatment phase early, before the maximum number of treatments are completed, if:

- The clinician feels the patient has achieved a sustained remission (QIDS SR 16 score <5 on two consecutive assessments) or the clinician determines additional treatments are not clinically warranted
- The patient has worsening depression, severe psychotic symptoms, or becomes suicidal

If these situations occur the patient should be scheduled for an End of Treatment Visit.

The patient can decide not to continue treatment for any reason. They should be encouraged to complete an End of Treatment Visit.

7.4 End of Treatment (EOT) Visit

Patients will complete an EOT visit within one week of their last study treatment. If patients do not stop treatment early, this will occur after the 9th ECT treatment or the 6th ketamine

treatment. If patients stop earlier, the EOT visit will occur within one week of this final treatment. Patients may continue to receive ECT or ketamine clinically, but the EOT visit marks the end of the acute treatment phase for the study.

Vitals
Update Medical History
Update Psychiatric Medication Log Evaluation for AEs or SAEs
Clinical Psychiatric Evaluation

Patient Rated Behavioral Scales
- QIDS SR 16 (to be completed first)
- GSE MY
- SMCQ
- PGI C & PGI I
- QOLS
- PRISE
- CPFQ

Clinician Rated Behavioral Scales
- MADRS
- CSSRS
- YMRS
- BPRS
- CGI S & CGI I
- CADSS

Diagnostic Interviews
- MINI
- ATHF

- Cognitive Assessments
  - MoCA
  - COWAT
  - HVLT R
  - Stroop

Patients will be classified as responders or non responders after the EOT Visit. Responders will be patients who had a ≥ 50% decrease in their QIDS SR 16 score from Baseline/Visit 1 to the EOT Visit. Non responders will have had < 50% decrease in their QIDS SR 16 score from Baseline/Visit 1 to the EOT Visit.

Responders will be asked to participate in the follow up phase of the study. Follow up visits will occur one month, three months, and six months after the EOT Visit.

7.5 Follow Up Visits for Responders

7.5.1 Month 1 Follow Up Visit (+/ 2 weeks)

Update Medical History
Update Psychiatric Medication Log Vitals (BP, heart rate, weight)
Evaluation for Adverse Events or Serious Adverse Events Psychiatric Evaluation

Patient Rated Behavioral Scales
- QIDS SR 16 (to be completed first)
- GSE MY
- SMCQ
- PGIC C & PGIC I
- QOLS
- PRISE
- CPFQ

Clinician Rated Behavioral Scales
- MADRS
- CSSRS
- YMRS
- BPRS
- CGI S & CGI I
- CADSS

- Cognitive Assessments
  - MoCA
  - COWAT
  - HVLT R
  - Stroop

7.5.2 Month 3 Follow Up Visit (+/ 2 weeks)

Update Medical History
Update Psychiatric Medication Log Vitals (BP, heart rate, weight)
Evaluation for Adverse Events or Serious Adverse Events Psychiatric Evaluation

Patient Rated Behavioral Scales

- QIDS SR 16 (to be completed first)
- GSE MY
- SMCQ
- PGIC C & PGIC I
- QOLS
- PRISE
- CPFQ

Clinician Rated Behavioral Scales

- MADRS
- CSSRS
- YMRS
- BPRS
- CGI S & CGI I
- CADSS

- Cognitive Assessments
  - MoCA
  - COWAT
  - HVLT R
  - Stroop

7.5.3 Month 6 Follow Up Visit (+/ 2 weeks)

Update Medical History
Update Psychiatric Medication Log Vitals (BP, heart rate, weight)
Evaluation for Adverse Events or Serious Adverse Events Psychiatric Evaluation

Patient Rated Behavioral Scales

- QIDS SR 16 (to be completed first)
- GSE MY
- SMCQ
- PGIC C & PGIC I
- QOLS
- PRISE
- CPFQ

Clinician Rated Behavioral Scales

- MADRS
- CSSRS
- YMRS
- BPRS
- CGI S & CGI I
- CADSS

- Cognitive Assessments
  - MoCA
  - COWAT
  - HVLT R
  - Stroop

7.6 Non Compliance

A patient can be withdrawn from the study for non compliance, per investigator discretion, if they miss two or more consecutive treatments during the acute treatment phase of the study.

7.7 Subject Withdrawal

Subjects may withdraw from study participation or from the study treatment at any time at their own request, or they may be withdrawn at any time at the discretion of the investigator for safety or behavioral reasons, or the inability of the subject to comply with the protocol required schedule of study visits or procedures at a given study site. If the subject elects to discontinue participation in the study or to discontinue study treatment, the investigator should:

- Inquire about the reason for withdrawal
- Request the subject to return for an EOT Visit to asses AEs / SAEs, safety endpoints, outcome events, vital status
- Follow up with the subject regarding any unresolved adverse events.

If the subject withdraws from the trial and also withdraws consent for disclosure of future information, no further evaluations should be performed and no additional data should be collected. Adequate documentation of this request should be obtained and retained in the subject's source file. True withdrawal of consent should be subject initiated and in writing. The sponsor may retain and continue to use any data collected before withdrawal of consent.

7.8 Lost to Follow Up

Contact information from the patient, including an emergency contact will be obtained at the time of screening. This information will be reviewed and verified at each clinic visit and telephone contact.

Patients will be considered lost to follow up after three attempts to contact. Study staff should document the attempts to contact in the research chart. Every attempt should be made to contact the patient as soon as possible after a missed visit. The site should access medical records, other health care professionals, institutional databases and any other means to contact the patient as allowed by their IRB. All attempts to contact the patient should be documented in the medical records. The patient's vital status should be obtained, if possible, at the time of the end of study (6 months follow up) from a reliable source or from medical records.

7.9 Study Termination

This study may be terminated or suspended at any time. If the study is terminated or suspended, the sponsor will promptly inform the investigators / institutions and PCORI. The IRB should be promptly informed and provided the reasons(s) for the termination or suspension by the sponsor and by the investigator / institution, as specified by the applicable regulatory requirement(s).

8. Safety Monitoring and Reporting

Patients will be closely monitored for adverse events (AEs) or serious adverse events (SAEs), including worsening of depression symptoms. Study investigators will be able to modify treatment or remove patients from the trial based on their clinical discretion and specific patient outcomes.

An adverse event is the development of an undesirable medical condition or the deterioration of a pre existing medical condition following or during exposure to a pharmaceutical product, whether or not considered causally related to the product. An undesirable medical condition can be symptoms (e.g., nausea, headache, fatigue, blurry vision) or signs (e.g., rapid or irregular heart rate, hypertension). In clinical studies, an AE can include an undesirable medical condition occurring at any time after the informed consent is signed even if no study treatment has been administered.

Assessment of adverse events, including grading of severity and attribution to research will start at the time of consent. AEs will be evaluated at each visit.

8.1 Unexpected Adverse Events

The following adverse events will be collected from the time of randomization for the study:

- Adverse events that are not listed in the current labeling for ketamine (Ketalar Product Information) or ECT. This includes events that are similar to those on the labeling but differ from the event because of greater severity or specificity.
- The following AEs should be captured and recorded on the AE form:
  - Prolonged seizure
  - Tardive seizure (late occurring)
  - Delirium (prolonged)
  - Psychosis (prolonged)

- Suicide Attempt
- Severe hypertension (prolonged)
- Dissociation (prolonged)
- Substance abuse (new onset/reoccurrence)
- Clinically significant arrhythmia
- Pregnancy

8.2 Study Treatment Discontinuation Adverse Events

Events that lead to the discontinuation of either treatment (ECT or ketamine) during the treatment phase, before completion of the final dose, will be collected for the study. These events will be recorded on either an AE or an SAE form.

8.3 Serious Adverse Events

All SAEs that meet the following definition will be collected from the time of consent until completion of either the EOT Visit (for non responders) or the six month follow up visit (for responders).

- Results in death
- Is immediately life threatening
- Requires in patient hospitalization or prolongation of existing hospitalization
- Results in persistent or significant disability/incapacity or substantial disruption of the ability to conduct normal life functions
- Is a congenital abnormality or birth defect
- Is an important medical event that may jeopardize the patient or may require medical intervention to prevent one of the outcomes listed above

8.4 Documentation and Reporting of Serious Adverse Events

SAEs will be reported to C5Research within 24 hours of learning of the event. The causality of the SAE (the relationship to the study treatment/procedures) will be assessed by the investigator. The SAE will also be documented on the appropriate eCRF.

Since the use of Ketamine in the ELEKT D study is exempted from IND reporting, the Investigator does not have the responsibility to report any AEs/SAEs to the FDA**.**

Pregnancy

All pregnancies should be reported to C5Research within 24 hours of becoming aware of the pregnancy.

- Maternal exposure If a patient becomes pregnant during the course of the study, study treatment will be continued or discontinued per investigator discretion. If any pregnancy occurs in the course of the study, the investigator must inform C5Research within 24 hours of awareness of the pregnancy.
- Paternal exposure There are no restrictions against fathering a child when receiving either ECT or ketamine treatment.

9. Statistical Plan

The key exposures of this study are alternate day ECT treatment and twice a week ketamine infusion. The primary analyses will be performed on the modified intention to treat (mITT) population defined as a randomized patient having at least one treatment and one valid QIDS SR16 measurement during the acute treatment phase. Percent of responders is the primary outcome in this study. A responder is defined as a subject with a ≥50% decrease from baseline in the primary endpoint (QIDS SR 16).

Multiple imputations may be implemented to achieve completeness of the data. In an unlikely case that missing data are non ignorable, pattern mixture modeling will be applied.

As a general principle the statistical analysis will follow the pre specified statistical analysis plan (SAP). The SAP will be finalized prior to the end of the study. The SAP will address how missing data will be handled.

The primary outcome measure of response rate will be compared between ketamine and ECT using a chi square test. A multivariable logistic regression model will be constructed, to account for potential heterogeneity of treatment effect caused by confounding variables. A similar analytic strategy will be applied to evaluate cognitive function and quality of life.

Sample size

The sample size justification will be for the primary outcome measure of response rate. Historical data reveal that the overall response rate as well as the respective response rate of ketamine and of ECT is around 50% 60% on various scale measurements in patients with treatment resistant depression. Assuming an observed difference of 10% and an acceptable difference margin of 5% with a 1 sided alpha=0.025, a sample of 400 patients (200 per group) provides 81.8% power to detect a treatment response, based on the Farrington Manning score test of risk difference. The total sample also considers a 10% attrition rate.

10. Study Committees

The following committees will be responsible for the management of the study and the monitoring of the safety of the study patients.

10.1 Executive Committee

The Executive Committee (EC) will have scientific responsibility for the study. They will review study conduct and progress, consider recommendations from the Data and Safety Monitoring Board (DSMB), and resolve any other study related issues. The EC will serve as the publishing committee for the study. The EC Charter document will guide the conduct of the EC.

10.2 Data and Safety Monitoring Board

A Data and Safety Monitoring Board (DSMB) will be appointed to monitor the key safety and efficacy outcomes at regular intervals, to safeguard the safety and interests of the study

participants, and maintain/uphold the scientific merit of the study. Members of the DSMB will not be investigators of the study. The DSMB Charter document will guide the conduct of the DSMB, and will include the procedures and stopping rules for the study.

10.3 Stakeholders Committee

A Stakeholder Advisory Committee will be formed with investigators, patient partners, patient advocacy groups (i.e. Ketamine Advocacy group, NAMI), third party payer representatives (i.e. Medical Mutual, Blue Cross). The committee will meet during the study to review study conduct and provide input on the progress of the study. The Stakeholder Committee will be involved in disseminating the final study results, information and other materials in lay language to patients/non scientists.

10.4 Reporting Plan

Study progress reports will be presented at regular intervals to the Executive Committee, the Stakeholders Committee, the Data and Safety Monitoring Board (DSMB) and to the Cleveland Clinic and site Institutional Review Boards (IRBs). Since this is an un blinded study, the total number of subjects enrolled in each treatment arm will be reported. The reports may include information on demographics, AEs/SAEs, significant protocol deviations, retention/withdrawals.

Table 5: Reporting Timeline

Committee	Participants	Suggested Frequency
Executive Committee	Lead PI, site PIs	Quarterly
Data Safety Monitoring Board	DSMB Members, P.I.	Twice / year
Stakeholders Committee	All Investigators and Consultants including Patient	Twice / year
Investigational Review Board	Cleveland Clinic and individual site IRBs	Annually or more as needed.

11. Data Handling and Record Keeping

11.1 Data Collection

Data will be collected by the study personnel at the site. Data sources include patient reports, questionnaires and available medical records. An eCRF must be completed for each randomized patient. It is the responsibility of the Investigator to ensure that the eCRF is completed accurately and in a timely manner. Screen failures should be recorded on the electronic screen failure log with the reason for ineligibility.

11.2 Retention of Records

The Investigators must maintain all confidential study documentation and take measures to prevent accidental or premature destruction of these documents. Documents should be retained for a minimum of six years after the completion or discontinuation of the clinical trial. However, applicable regulatory and institutional requirements will be taken into account in the event that a longer period is required.

12. Study Monitoring, Auditing, and Inspecting

Study Monitoring Plan

C5Research is responsible for monitoring the conduct of this study. The study will be monitored according to the Monitoring Plan, and per the applicable C5Research Standard Operation Procedures for clinical monitoring. It is the responsibility of the Investigator to allocate adequate time for monitoring, to allow the monitor to access the medical records of the patients and to provide for adequate space to conduct the monitoring visit.

13. Ethical Considerations

The study protocol, consent forms, data collection forms, and recruitment materials, if applicable, will be submitted to each site’s IRB. All study personnel will have completed training in the Protection of Human Subjects according to Institutional guidelines.

Institutional Review Board (IRB)

It is the investigator’s responsibility to ensure that the study protocol and informed consent documents are reviewed and approved by the appropriate IRB. Each clinical site will obtain a letter of approval from the IRB before approaching participants. Sites will provide C5Research with copies of the initial IRB approval notice prior to enrolling the first patient, and subsequent renewals, as well as copies of the IRB approved consent and other IRB approved forms.

If, during the study, it is necessary to amend either the protocol or informed consent document, the investigator will be responsible for ensuring that the IRB reviews and approves the amended documents. IRB approval of any procedures must be obtained before implementing new processes or procedures.

Informed Consent Document and Process

All subjects for this study will be provided a consent form describing this study and providing sufficient information for subjects to make an informed decision. The informed consent document will inform patients of their right to refuse any release of their protected health information. Each clinical site, according to local IRB requirements, is allowed to modify this informed consent document and make any necessary editorial changes, as long as neither the meaning nor intent of any section is changed.

The investigator or his/her designee (i.e., research coordinator or study nurse) will inform the patient of all aspects of the study pertaining to the patient’s participation in it. The process for obtaining informed consent will be in accordance with all applicable regulatory requirements. The informed consent form (ICF) must be signed and dated by the patient and the investigator or his/her designee BEFORE the patient can participate in the study. The participant will receive a copy of all signed and dated documents, and the originals will be retained in the patient’s study file or medical record.

Subject Information and Consent

Each clinical site is responsible for the confidentiality of the data associated with participants enrolled in this study, in the same manner that it is responsible for the confidentiality of any patient information within its sphere of responsibility. All forms used for the study data will be identified by coded identification number, which will be generated at the clinical center, to maintain subject confidentiality. All records will be kept in locked file cabinets at the clinical centers with access limited to study staff, and all study staff will identify participants via their unique identifier. Clinical information will not be released without written permission of the participant, except as necessary for monitoring by the IRB or DSMB. The participant grants permission to share research data with these entities in the consent document. Federal regulations govern the protection of patients rights relative to data confidentiality and use of research data.

Consent procedures and forms, and the communication, transmission and stoppage of patient data will comply with individual site IRB requirements for compliance with The Health Insurance Portability and Accountability Act (HIPAA). The Privacy Rule of HIPAA governs the protection of an individuals identifiable health information. C5Research will ensure that clinical centers associated with the project comply with HIPAA regulations by requiring documentation from the IRBs with the appropriate authorization or consent form. C5Research will maintain copies of all relevant documents from each clinical center. If IRB approvals are not current, data will not be accepted by C5Research. A secure, electronic data management system will be used to ensure the confidentiality of electronic protected health information. All questionnaires and study related materials will be labeled with each participants coded identification number; there will be no protected health information indicated on the forms.

14. Publication and Disclosure

The study findings will be disseminated to the public through manuscripts, scientific and patient organization led conferences, press releases and through a dedicated website. The PIs may also publicize the study findings through talks and public symposia with their local mental health advocacy organizations (NAMI, DBSA, etc.).

This study will be registered on ClinicalTrials.gov.

15. References

1. Global Burden of Disease; 2004. Geneva, Switzerland: World Health Organization, 2008.
2. Fournier JC, DeRubeis RJ, Hollon SD, et al. Antidepressant drug effects and depression severity: A patient level meta analysis. JAMA. 2010;303(1); 47 53
3. Turner EH, Matthews AM, Linardatos E, Tell RA, Rosenthal R. Selective Publication of Antidepressant Trials and Its Influence on Apparent Efficacy. New England Journal of Medicine. 2008;358(3):252 60.
4. Rush AJ, Trivedi MH, Wisniewski SR, Nierenberg AA, Stewart JW, Warden D, et al. Acute and longer term outcomes in depressed outpatients requiring one or several treatment steps: a STAR*D report. American Journal of Psychiatry. 2006;163(11):1905 17.
5. Trivedi MH, Rush AJ, Wisniewski SR, Nierenberg AA, Warden D, Ritz L, et al. Evaluation of outcomes with citalopram for depression using measurement based care in STAR*D: implications for clinical practice. American Journal of Psychiatry. 2006;163(1):28 40.
6. Mrazek DA, Hornberger JC, Altar CA, Degtiar I. A Review of the Clinical, Economic, and Societal Burden of Treatment Resistant Depression: 1996 2013. Psychiatric Services. 2014;65(8):977 87.
7. Nemeroff CB. Prevalence and management of treatment resistant depression. J Clin Psychiatry. 2007;68 Suppl8:17 25.
8. Crown WH, Finkelstein S, Berndt ER, Ling D, Poret AW, Rush AJ, et al. The impact of treatment resistant depression on health care utilization and costs. The Journal of Clinical Psychiatry. 2002;63(11);963 71.
9. Kellner CH, Greenberg RM, Murrough JW, Bryson EO, Briggs MC, Pasculli RM. ECT in Treatment Resistant Depression. American Journal of Psychiatry. 2012;169(12):1238 44.
10. The UKECTRG. Efficacy and safety of electroconvulsive therapy in depressive disorders: a systematic review and meta analysis. The Lancet. 2003;361(9360):799 808.
11. Lisanby SH. Electroconvulsive Therapy for Depression. New England Journal of Medicine. 2007;357(19):1939 45.
12. Golden, C. J., & Freshwater, S. M. (1978). Stroop color and word test.
13. Blair, J. R., & Spreen, O. (1989). Predicting premorbid IQ: a revision of the National Adult Reading Test. The Clinical Neuropsychologist, 3(2), 129 136.
14. aan het Rot M, Collins KA, Murrough JA, Perez AM, Reich DL, Charney DS, et al. Safety and efficacy of repeated dose intravenous ketamine for treatment resistant depression. Biological psychiatry. 2010;67(2):139 45.
15. Newport DJ, Carpenter LL, McDonald WM, Potash JB, Tohen M, Nemeroff CB. Ketamine and Other NMDA Antagonists: Early Clinical Trials and Possible Mechanisms in Depression. The American journal of psychiatry. 2015;172(10):950 66.
16. van Waarde JA, van Oudheusden LJ, Verway B, Giltay EJ, van der Mast RC. European archives of psychiatry and clinical neuroscience. 2013 Mar;263(2):167 75. Doi: 10.1007/s00406 012 0342 7.
17. Lapidus KA, Kellner CH. Journal of ECT. 2011 Sep;27(3):244 6. Doi: 10.1097/YCT.0b013e31820059e1.

16. Appendix

Table 1 Outcome Measures and Scales		
MEASURE	NAME	DESCRIPTION
**DIAGNOSTIC INTERVIEW**		
MINI 7.0	Mini Neuropsychiatric Interview	Diagnostic interview used to determine DSM 5 diagnosis (30 mins)
ATHF	Antidepressant Treatment History Form	Records dosage and duration of antidepressants and other psychiatric medications (30 mins)
**PATIENT RATED SCALES**		
QIDS SR 16 (Primary OutcomeMeasure)	Quick Inventory of Depressive Symptoms	Self report of depressive symptoms based on DSM diagnostic criteria (10 mins)
GSE My	Global Self Evaluation of Memory	Self reported scale of global memory (2 mins)
SMCQ	Squire Memory Complaint Questionnaire	Self report of memory issues before and after ECT (5 mins)
PGI C and PGI I	Patient Global Impression Scale for Severity and Improvement for Depression	7 point scales assessing improvement and severity of depression(2 mins)
QOLS	Quality of Life Scale	Self reported questionnaire that measures quality of life in 8 domains (5 mins)
PRISE	Patient Rated Inventory of Side Effects	Self report of adverse events specific to nine organ or function systems (<5 mins)
**CLINICIAN RATED SCALES**		
MADRS	Montgomery Asberg Depression Rating Scale	Measures severity of depression symptoms including sadness, concentration, sleep, and disruptive thoughts (10 20 mins)
CSSRS	Columbia Suicide Severity Rating Scale	Assessment of suicidal ideation (10 mins)
CADSS*	Clinician Administered Dissociative Symptoms Scale	Dissociative symptom scale to be administered post treatment (10 mins)
YMRS	Young Mania Rating Scale	Measures symptoms of mania (10 mins)
BPRS*	Brief Psychiatric Rating Scale	Measures positive symptoms of psychosis (5 mins)
CGI S and CGI I	Clinical Global Impression Scale for Severity and Improvement	Scales to record global clinical impression by a clinician regarding improvementand severity of patients mental condition (5 mins)
**COGNITIVE TESTING**		
MoCA	Montreal Cognitive Assessment	Tests cognitive function covering 8 cognitivedomains including visuospatial assessment, short term memory and workingmemory (10 mins)
COWAT	Controlled Oral Word Association Test	Verbal fluency test that measures spontaneous production of words belonging tothe same category or beginning with the same letter (5 10 mins)
HVLT R	Hopkins verbal Learning Test Revised	Verbal learning and memory test with six alternate forms (10 mins)
Stroop	Stroop Color Word Test	Measures processing speed and selective inhibition (5 mins)
NAART	North American Adult Reading Test	Estimate of premorbid intellectual ability (3 mins)
CPFQ	Cognitive and Physical Functioning	Assessment of motivation, energy level, & mental acuity (5 mins)
*To be administered post treatment		

**Visit**	**Screening** (within 28 days after clinical ECT referral)	**Randomization/** (Up to 1 week after Screening Visit)	**Treatment Phase (Up to 9 treatments over 3- 5 weeks)**									**EOT** (up to 1 week after last treatment)
			Baseline/ V1	V2	V3	V4	V5	V6	V7	V8	V9	V10
Informed consent	X											
Eligibility Criteria	X											
Demographics	X											
Medical history	X		X									
Urine pregnancy test	X											
Vitals (BP, heart rate, height, weight)			X	X	X	X	X	X	X	X	X	X
Randomization		X										
Psychiatric Evaluation	X		X	X	X	X	X	X	X	X	X	X
ECT			X	X	X	X	X	X	X	X	X	
Psychiatric Medication Log			X	X	X	X	X	X	X	X	X	X
Somatic Therapies Log			X	X	X	X	X	X	X	X	X	X
Serious Adverse Events/AEs			X	X	X	X	X	X	X	X	X	X
**Diagnostic Interview**												
MINI 7.0	X											
ATHF	X											
**Patient Rated Scales**												
QIDS-SR-16			X	X		X		X	X		X	X

GSE-MY					X	X		X	X		X	X
SMCQ			X		X	X		X	X		X	X
PGI-C			X		X	X		X	X		X	X
PGI-I					X	X		X	X		X	X
QOLS			X		X	X		X	X		X	X
PRISE			X		X	X		X	X		X	X
CPFQ			X		X	X		X	X		X	X
**Clinician Rated Scales**												
MADRS	X		X		X	X		X	X		X	X
CSSRS			X		X	X		X	X		X	X
YMRS	X		X		X	X		X	X		X	X
BPRS*			X		X	X		X	X		X	X
CGI-S**			X		X	X		X	X		X	X
CGI-I**					X	X		X	X		X	X
CADSS*			X		X	X		X	X		X	X
**Cognitive Assessments**												
MoCA	X											X
COWAT			X									X
HVLT-R			X									X
Stroop			X									X
NAART			X									

*CADSS and BPRS to be administered post treatment.

**CGI-S and CGI-I to be performed by psychiatrist.

941

**Visit**	**Screening** **(Within 28 days after clinical ECT referral)**	**Randomization** **(Up to 1 week from Screening Visit)**	**Treatment Phase** **(Up to 6 treatments over 3- 5 weeks)**						**EOT** **(+/- 1 week)**
			Baseline/V1	V2	V3	V4	V5	V6	V10
Informed consent	X								
Eligibility Criteria	X								
Demographics	X								
Medical history	X		X						
Urine pregnancy test	X								
Vitals (BP, heart rate, height, weight)			X	X	X	X	X	X	X
Randomization		X							
Psychiatric Evaluation	X		X	X	X	X	X	X	X
Ketamine Infusion			X	X	X	X	X	X	
Psychiatric Medication Log			X	X	X	X	X	X	X
Somatic Therapies Log			X	X	X	X	X	X	X
Serious Adverse Events/AEs			X	X	X	X	X	X	X
**Diagnostic Interview**									
MINI 7.0	X								
ATHF	X								
**Patient Rated Scales**									
QIDS-SR-16			X	X	X	X	X	X	X

GSE-MY				X	X	X	X	X	X
SMCQ			X	X	X	X	X	X	X
PGI-C			X	X	X	X	X	X	X
PGI-I				X	X	X	X	X	X
QOLS			X	X	X	X	X	X	X
PRISE			X	X	X	X	X	X	X
CPFQ			X	X	X	X	X	X	X
**Clinician Rated Scales**									
MADRS	X		X	X	X	X	X	X	X
CSSRS			X	X	X	X	X	X	X
YMRS	X		X	X	X	X	X	X	X
BPRS*			X	X	X	X	X	X	X
CGI-S**			X	X	X	X	X	X	X
CGI-I**				X	X	X	X	X	X
CADSS*			X	X	X	X	X	X	X
**Cognitive Assessments**									
MoCA	X								X
COWAT			X						X
HVLT-R			X						X
Stroop			X						X
NAART			X						

*CADSS and BPRS to be administered post treatment.

**CGI-S and CGI-I to be performed by psychiatrist.

Visit	**Month 1** (+/- 2 weeks)	**Month 3** (+/- 2 weeks)	**Month 6** (+/- 2 weeks)
Update Medical History	X	X	X
Vitals (BP, heart rate, weight)	X	X	X
Psychiatric Evaluation	X	X	X
Psychiatric Medication Log	X	X	X
Somatic Therapies Log	X	X	X
Serious Adverse Events/AEs	X	X	X
**Patient Rated Scales**			
QIDS-SR-16	X	X	X
GSE-MY	X	X	X
SMCQ	X	X	X
PGI-C & PGI-I	X	X	X
QOLS	X	X	X
PRISE	X	X	X
CPFQ	X	X	X
**Clinician Rated Scales**			
MADRS	X	X	X
CSSRS	X	X	X
YMRS	X	X	X
BPRS	X	X	X
CGI-S & CGI-I*	X	X	X
CADSS	X	X	X
**Cognitive Assessments**			
MoCA	X	X	X
COWAT	X	X	X
HVLT-R	X	X	X
Stroop	X	X	X

*To be performed by psychiatrist
