# Supplementary material for: Ketamine vs Electroconvulsive Therapy for Treatment-Resistant Depression: A Secondary Analysis of a Randomized Clinical Trial
Source: JAMA Netw Open. 2024 Jun 25;7(6):e2417786. doi: 10.1001/jamanetworkopen.2024.17786 (PMC11200139; doi:10.1001/jamanetworkopen.2024.17786)
Supplement: Supplement 2. — eFigure 1. CONSORT Diagram of ELEKT-D Participants Included in This Report eFigure 2. Changes in QIDS-SR16 With Ketamine vs ECT Based on Hospital Admission Status at the Time of First Treatment eFigure 3. Differential Rates of Response and Remission With Ketamine vs ECT Based on Standardized Scores on the North American Adult Reading Test 35 (NAART-35) eFigure 4. Treatment Effects of Ketamine vs ECT Based on MADRS Stratified by Less (Left Panel) or More (Right Panel) Severe Baseline Depression Severity eFigure 5. Treatment Effects of ECT Based on Presence of Comorbid PTSD eFigure 6. Treatment Effects of ECT Based on HVLT-R Levels at Baseline eFigure 7. Treatment Effects of Ketamine and ECT Based on Body Mass Index (BMI) Categories eTable 1. Baseline Feature of ELEKT-D Participants Randomized to ECT Who Dropped Out (ie, Did Not Complete Any Posttreatment Assessment) vs Those Who Did Not eTable 2. Baseline Features That Predict Response and Remission With ECT vs Ketamine eTable 3. Baseline Features That Predict Response and Remission in Separate Analyses for ECT and Ketamine Treatment Groups [file jamanetwopen-e2417786-s002.pdf]

Supplemental Online Content

Jha MK, Wilkinson ST, Krishnan K, et al. Ketamine vs electroconvulsive therapy for treatment-resistant depression: a secondary analysis of a randomized clinical trial. *JAMA Netw Open*. 2024;7(6):e2417786. doi:10.1001/jamanetworkopen.2024.17786

eFigure 1. CONSORT Diagram of ELEKT-D Participants Included in This Report
eFigure 2. Changes in QIDS-SR16 With Ketamine vs ECT Based on Hospital Admission Status at the Time of First Treatment
eFigure 3. Differential Rates of Response and Remission With Ketamine vs ECT Based on Standardized Scores on the North American Adult Reading Test 35 (NAART-35)
eFigure 4. Treatment Effects of Ketamine vs ECT Based on MADRS Stratified by Less (Left Panel) or More (Right Panel) Severe Baseline Depression Severity
eFigure 5. Treatment Effects of ECT Based on Presence of Comorbid PTSD
eFigure 6. Treatment Effects of ECT Based on HVLT-R Levels at Baseline
eFigure 7. Treatment Effects of Ketamine and ECT Based on Body Mass Index (BMI) Categories
eTable 1. Baseline Feature of ELEKT-D Participants Randomized to ECT Who Dropped Out (ie, Did Not Complete Any Posttreatment Assessment) vs Those Who Did Not
eTable 2. Baseline Features That Predict Response and Remission With ECT vs Ketamine
eTable 3. Baseline Features That Predict Response and Remission in Separate Analyses for ECT and Ketamine Treatment Groups

This supplemental material has been provided by the authors to give readers additional information about their work.

© 2024 Jha MK et al. *JAMA Network Open*.

eFigure 1. CONSORT Diagram of ELEKT-D Participants Included in This Report

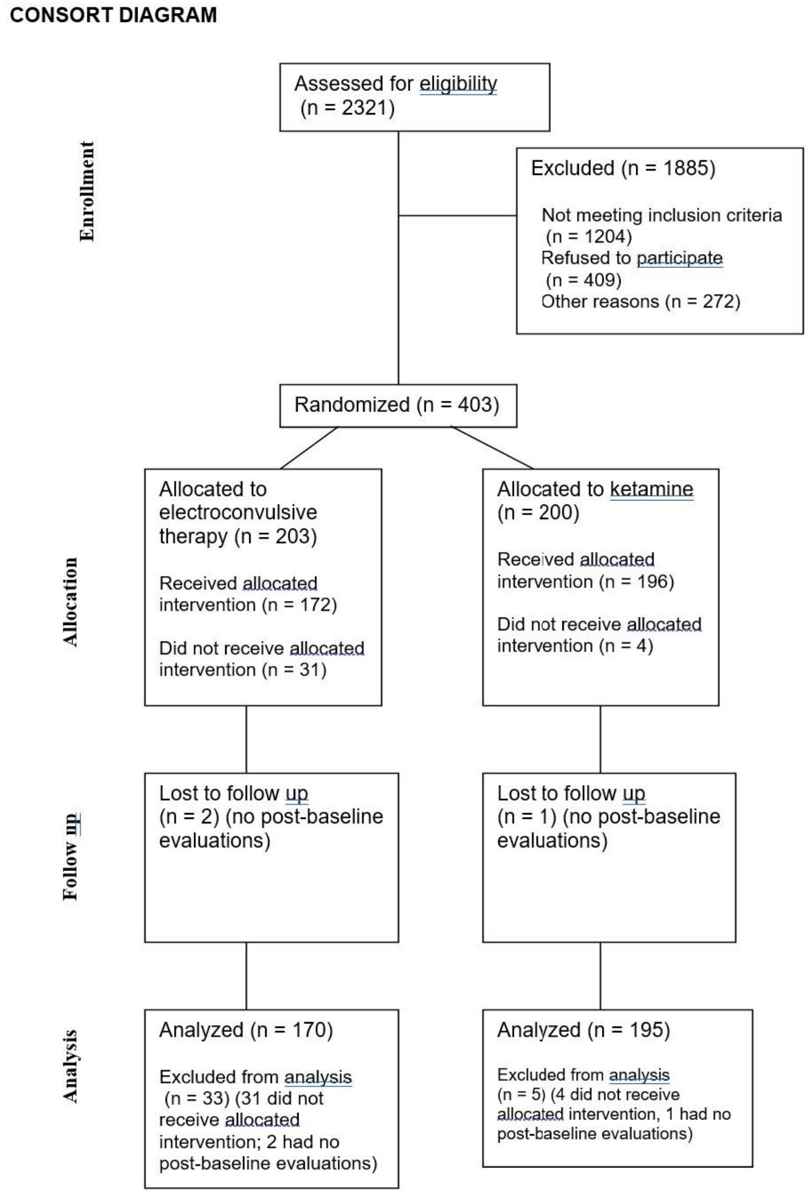

© 2024 Jha MK et al. *JAMA Network Open*.

eFigure 2. Changes in QIDS-SR16 With Ketamine vs ECT Based on Hospital Admission Status at the Time of First Treatment

Panel A. Levels of QIDS-SR16 from baseline to the end-of-treatment visit.

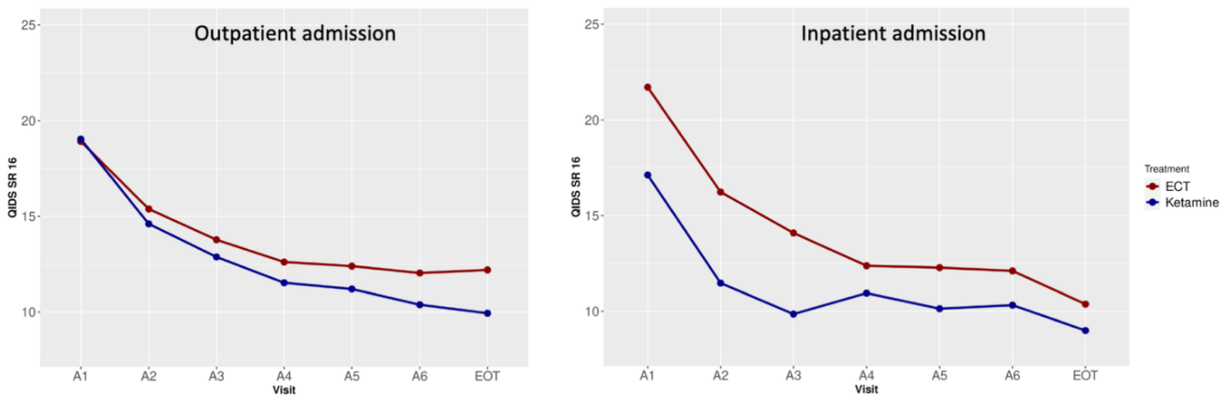

Panel B. Changes in QIDS-SR16 from baseline to end-of-treatment visit.

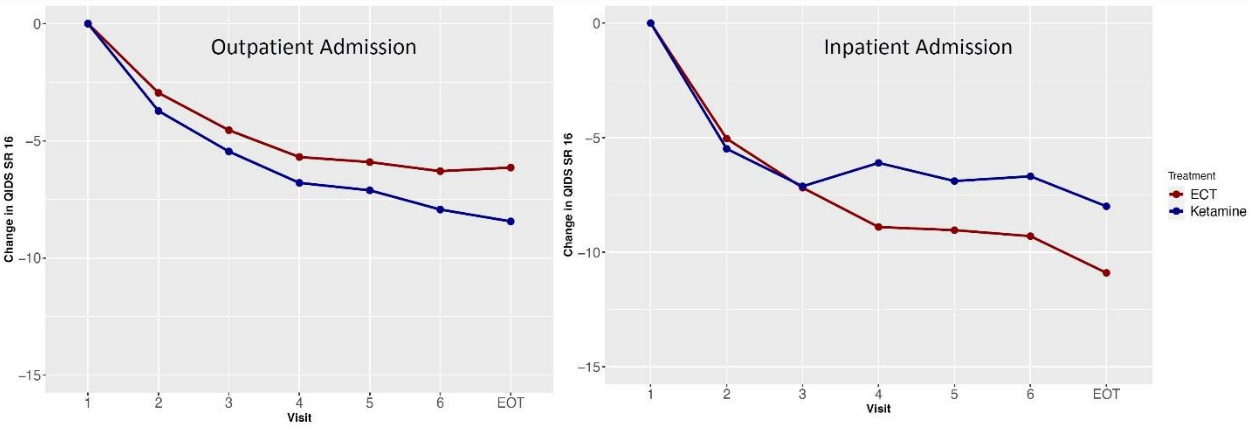

Legends: ECT is electroconvulsive therapy, QIDS-SR16 is 16-item Quick Inventory of Depressive Symptomatology Self-Report version. A1 to A6 are visits 1 to 6 and EOT is end-of-treatment visit.

© 2024 Jha MK et al. *JAMA Network Open*.

eFigure 3. Differential Rates of Response and Remission With Ketamine vs ECT Based on Standardized Scores on the North American Adult Reading Test 35 (NAART-35)

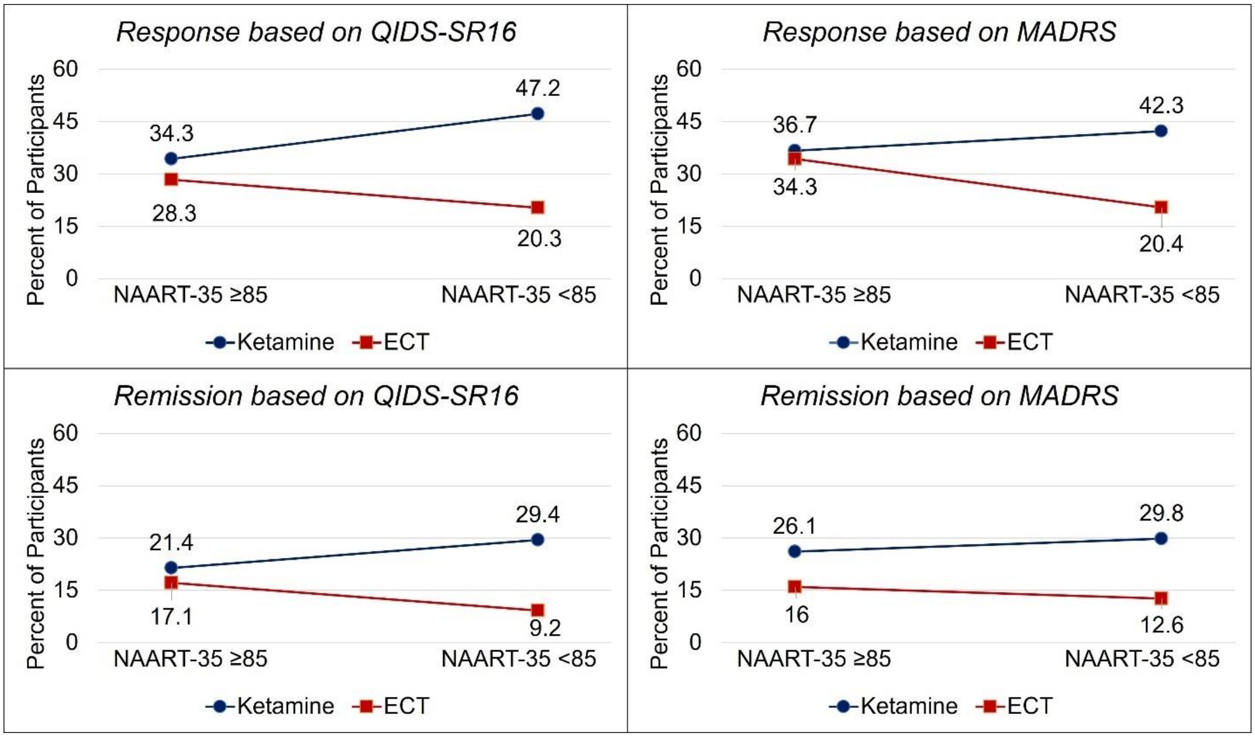

Legends: ECT is electroconvulsive therapy, QIDS-SR16 is 16-item Quick Inventory of Depressive Symptomatology Self-Report version.

© 2024 Jha MK et al. *JAMA Network Open*.

eFigure 4. Treatment Effects of Ketamine vs ECT Based on MADRS Stratified by Less (Left Panel) or More (Right Panel) Severe Baseline Depression Severity

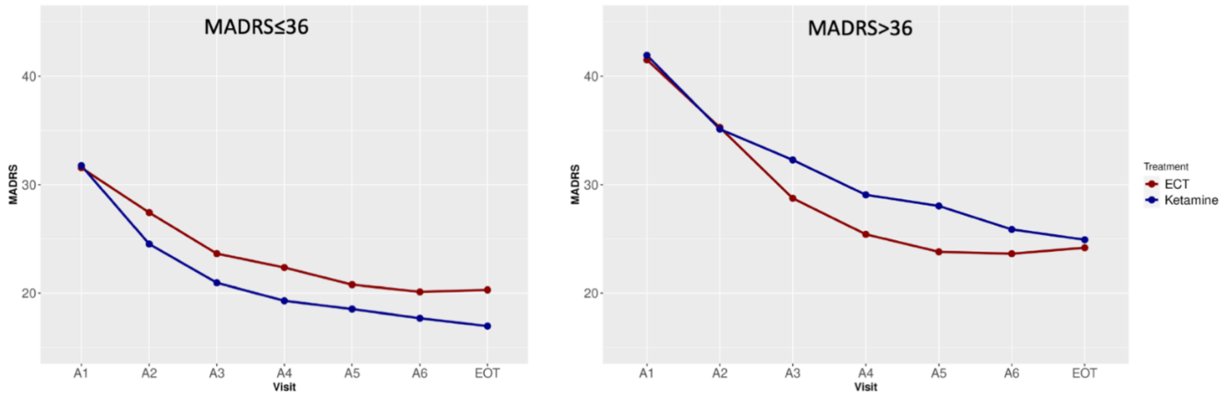

Legends: MADRS is Montgomery Åsberg Depression Rating Scale. Least square means from mixed model analyses were plotted for both treatment groups [electroconvulsive therapy (ECT) or ketamine] based on baseline depression severity thresholds of moderate severe or severe (MADRS ≤36) or very severe (MADRS >36).

© 2024 Jha MK et al. *JAMA Network Open*.

eFigure 5. Treatment Effects of ECT Based on Presence of Comorbid PTSD

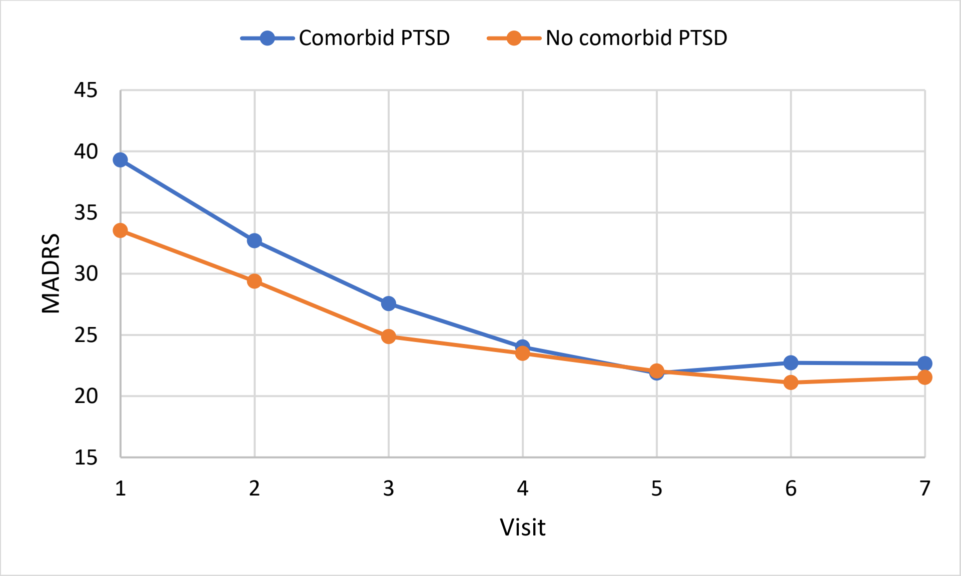

Legends: ECT is electroconvulsive therapy, PTSD is post-traumatic stress disorder. MADRS is Montgomery Åsberg Depression Rating Scale. Visit 7 was the end-of-treatment visit.

© 2024 Jha MK et al. *JAMA Network Open*.

eFigure 6. Treatment Effects of ECT Based on HVLT-R Levels at Baseline

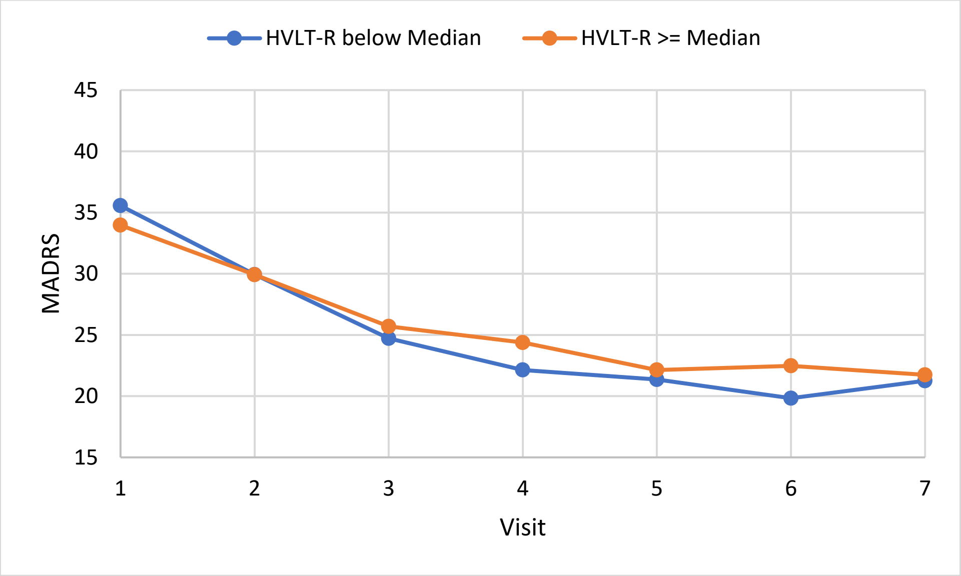

Legends: ECT is electroconvulsive therapy, HVLT is the Hopkins Verbal Learning Test. MADRS is Montgomery Åsberg Depression Rating Scale. Visit 7 was the end-of-treatment visit.

© 2024 Jha MK et al. *JAMA Network Open*.

eFigure 7. Treatment Effects of Ketamine and ECT Based on Body Mass Index (BMI) Categories

Panel A. Treatment effect of ketamine on changes in QIDS-SR16 based on BMI categories.

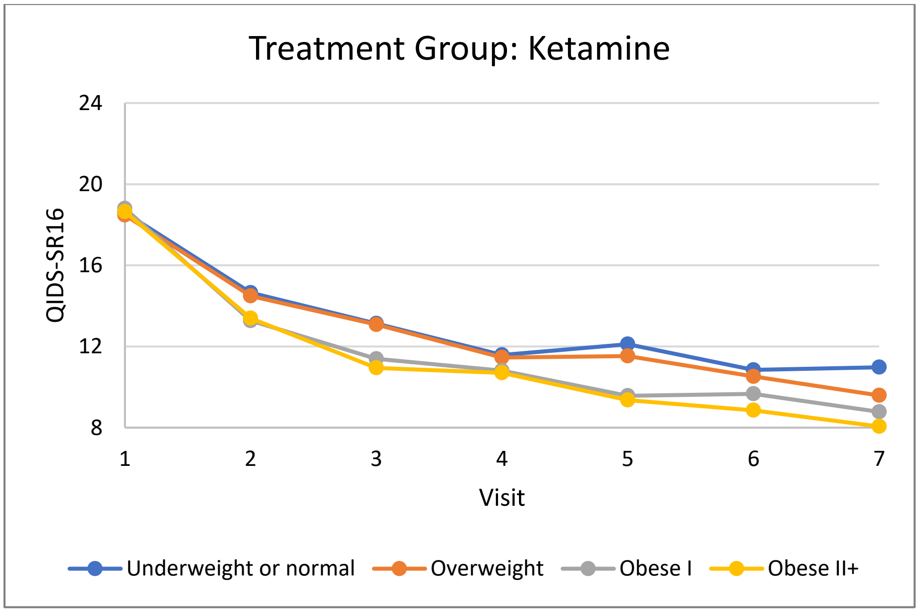

Panel B. Treatment effects of ketamine on changes in MADRS based on BMI categories.

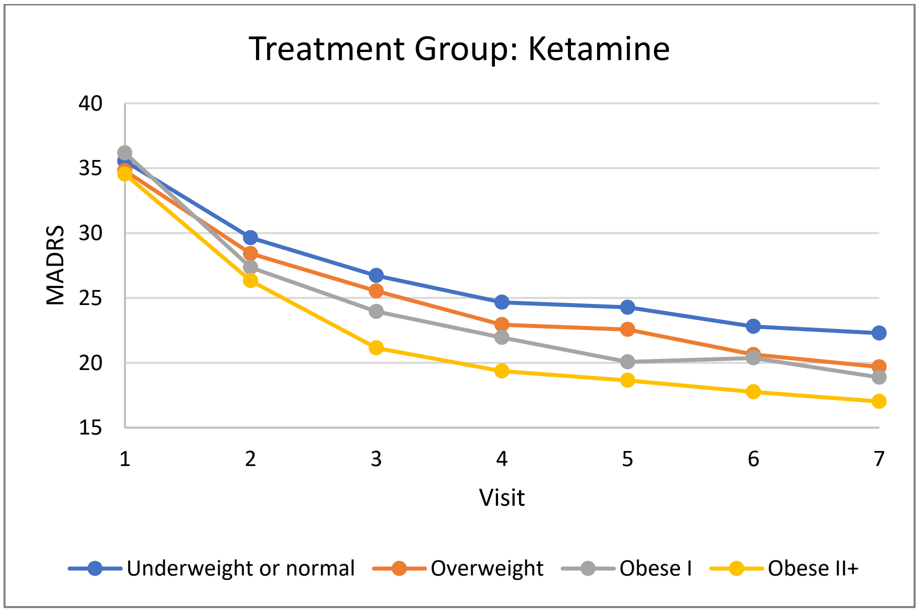

Panel C. Treatment effects of ECT on changes in QIDS-SR based on BMI categories.

© 2024 Jha MK et al. *JAMA Network Open*.

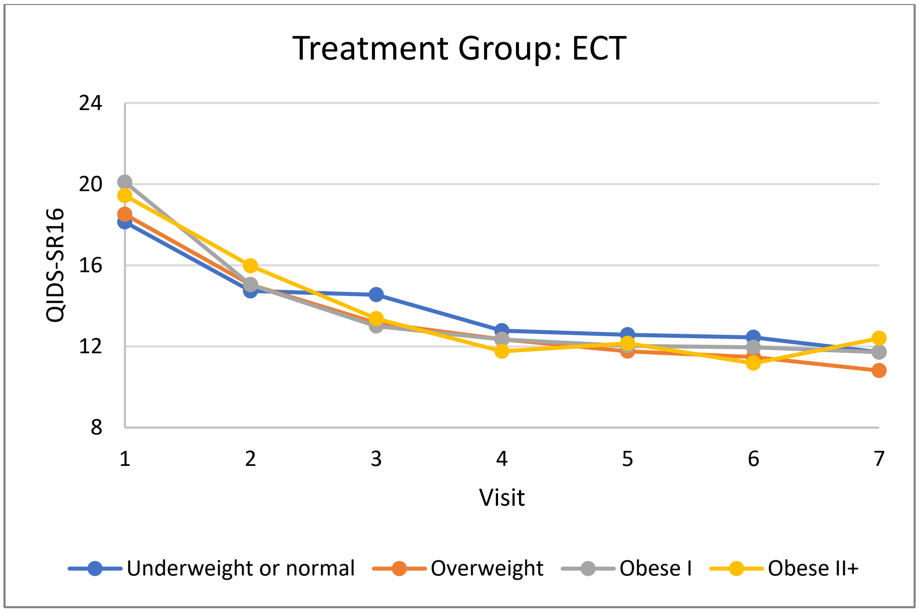

Legend: Categories of underweight or normal, overweight, obese I, and obese II+ were defined as BMI values of <25.0, 25.0 to 29.9, 30.0 to 34.9, and ≥35.0, respectively. QIDS-SR16 is 16-, item Quick Inventory of Depressive Symptomatology Self-Report version MADRS is Montgomery Åsberg Depression Rating Scale. Visit 7 was the end-of-treatment visit.

© 2024 Jha MK et al. *JAMA Network Open*.

eTable 1. Baseline Feature of ELEKT-D Participants Randomized to ECT Who Dropped Out (ie, Did Not Complete Any Posttreatment Assessment) vs Those Who Did Not

	Dropout from ECT	Did not drop out from ECT
Number	33	170
Categorical variables		
Male sex (%)	20 (60.6)	83 (48.8)
Female sex (%)	13 (39.4)	87 (51.2)
Race		
African descendant (%) new_race (%)	2 (6.1)	10 (5.9)
European descendant (%)	31 (93.9)	151 (88.8)
Other* (%)	0 (0.0)	9 (5.3)
Ethnicity		
Hispanic (%)	4 (12.1)	7 (4.1)
Non-Hispanic (%)	29 (87.9)	163 (95.9)
History of suicide attempt (%)	14 (42.4)	70 (41.2)
Anxious features absent	13 (39.4)	80 (47.1)
Anxious features present	20 (60.6)	90 (52.9)
No comorbid Post-traumatic Stress Disorder (%)	26 (78.8)	127 (74.7)
Comorbid Post-traumatic Stress Disorder (%)	7 (21.2)	43 (25.3)
No concurrent use of benzodiazepines (%)	26 (78.8)	114 (67.1)
Concurrent use of benzodiazepines (%)	7 (21.2)	56 (32.9)
No concurrent use of atypical antipsychotics (%)	22 (66.7)	123 (72.4)
Concurrent use of atypical antipsychotics (%)	11 (33.3)	47 (27.6)

© 2024 Jha MK et al. *JAMA Network Open*.

Initiation of treatment as outpatient (%)	33 (100.0)	149 (87.6)
Initiation of treatment as inpatient	0 (0.0)	21 (12.4)
Site		
Baylor College of Medicine (%)	12 (36.4)	36 (21.2)
Cleveland Clinic (%)	7 (21.2)	51 (30.0)
Mount Sinai (%)	6 (18.2)	28 (16.5)
Yale (%)	2 (6.1)	37 (21.8)
Johns Hopkins (%)	6 (18.2)	18 (10.6)
Continuous variables		
Age in years, Mean (SD)	49.85 (13.49)	46.56 (14.14)
QIDS-SR16, Mean (SD)	17.00 (4.24)	18.24 (4.17)
MADRS, Mean (SD)	31.00 (1.41)	32.64 (6.08)
MoCA, Mean (SD)	26.09 (2.49)	26.54 (2.59)
HVLT Delayed Recall, Mean (SD)	38.00 (5.66)	37.82 (14.67)
NAART-35 score, Mean (SD)	87.00 (11.31)	89.45 (8.75)
Body mass index (in kg/m^2), Mean (SD)	26.76 (8.46)	30.46 (7.91)

*including Asian, American Indian/native, multi-race and other races that were self-reported. ELEKT-D is the ELEctroconvulsive therapy (ECT) vs. Ketamine in patients with Treatment-resistant Depression study, ECT is Electroconvulsive Therapy, PTSD is post-traumatic stress disorder, QIDS-SR16 is 16-item Quick Inventory of Depressive Symptomatology Self-Report version, MADRS is Montgomery Åsberg Depression Rating Scale, MoCA is Montreal Cognitive Assessment, HVLT is Hopkins Verbal Learning Test, and NAART-35 is the North American Adult Reading Test 35.

© 2024 Jha MK et al. *JAMA Network Open*.

eTable 2. Baseline Features That Predict Response and Remission With ECT vs Ketamine

	QIDS-SR outcome		MADRS outcome	
	χ^2	p value	χ^2	p value
Response as the outcome				
NAART-35 standard score	5.92	0.015	4.61	0.032
Baseline depression severity*	0.38	0.54	1.46	0.23
MoCA score	1.87	0.17	0.13	0.72
HVLT-R Delayed Recall T-score	0.15	0.69	0.38	0.54
Concurrent benzodiazepine	2.37	0.12	1.55	0.21
Concurrent atypical antipsychotic	0.69	0.41	0.023	0.88
Body mass index	2.91	0.088	2.16	0.14
History of suicide attempt	0.0056	0.94	0.011	0.92
Inpatient status at first treatment	1.95	0.16	0.82	0.37
Presence of anxious features	0.025	0.88	0.77	0.38
Comorbid PTSD Diagnosis	1.14	0.28	0.32	0.57
Remission as the outcome				
NAART-35 standard score	6.17	0.013	1.89	0.17
Baseline depression severity*	0.29	0.59	0.021	0.89
MoCA score	0.50	0.48	0.41	0.52
HVLT-R Delayed Recall T-score	0.12	0.73	0.17	0.68
Concurrent benzodiazepine	1.37	0.24	0.69	0.41
Concurrent atypical antipsychotic	1.77	0.18	5.50	0.019
Body mass index	2.21	0.14	0.79	0.37

© 2024 Jha MK et al. *JAMA Network Open*.

History of suicide attempt	0.053	0.82	0.10	0.75
Inpatient status at first treatment	0.011	0.92	0.32	0.57
Presence of anxious features	0.036	0.85	0.55	0.46
Comorbid PTSD Diagnosis	0.91	0.34	0.075	0.78

Footnotes: * same measures as the outcome measure. There were no p <0.05 false discovery rate (FDR) correction for multiple comparisons. PTSD is post-traumatic stress disorder, QIDS-SR16 is 16-item Quick Inventory of Depressive Symptomatology Self-Report version, MADRS is Montgomery Åsberg Depression Rating Scale, MoCA is Montreal Cognitive Assessment, HVLT is Hopkins Verbal Learning Test, and NAART-35 is the North American Adult Reading Test 35.

© 2024 Jha MK et al. *JAMA Network Open*.

eTable 3. Baseline Features That Predict Response and Remission in Separate Analyses for ECT and Ketamine Treatment Groups

	Response as the outcome				Remission as the outcome			
	QIDS-SR16		MADRS		QIDS-SR16		MADRS	
	Odds Ratio**	95% CL	Odds Ratio	95% CL	Odds Ratio	95% CL	Odds Ratio	95% CL
ECT as the treatment								
Baseline depression severity*	0.98	0.70, 1.38	0.93	0.64, 1.33	0.62	0.41, 0.93	0.68	0.44, 1.05
MoCA score	1.29	0.91, 1.82	1.05	0.75, 1.48	0.98	0.65, 1.48	0.92	0.62, 1.36
HVLT Delayed Recall score	1.10	0.78, 1.56	0.92	0.65, 1.29	0.96	0.64, 1.45	0.97	0.65, 1.44
Concurrent benzodiazepine	0.79	0.39, 1.62	0.62	0.30, 1.29	0.51	0.20, 1.33	0.51	0.21, 1.25
Concurrent atypical antipsychotic	1.20	0.56, 2.57	1.40	0.65, 3.03	0.47	0.16, 1.40	0.37	0.13, 1.08
Body mass index	0.96	0.68, 1.34	1.03	0.74, 1.44	1.19	0.79, 1.78	1.00	0.68, 1.48
History of suicide attempt	1.03	0.53, 2.00	1.00	0.51, 1.96	1.13	0.51, 2.51	0.88	0.41, 1.89
Inpatient status at first treatment	3.67	1.28,10.49	2.36	0.82, 6.78	1.35	0.38, 4.76	1.95	0.64, 5.95
Presence of anxious features	0.61	0.30, 1.23	0.41	0.19, 0.85	0.53	0.23, 1.24	0.73	0.33, 1.63
Comorbid PTSD Diagnosis	0.85	0.37, 1.95	1.29	0.57, 2.94	1.02	0.38, 2.72	1.16	0.46, 2.94
Ketamine as the treatment								
Baseline depression severity*	0.91	0.67, 1.23	0.95	0.69, 1.31	0.49	0.35, 0.70	0.70	0.49, 0.99
MoCA score	0.98	0.71, 1.35	1.12	0.82, 1.53	1.09	0.77, 1.54	1.06	0.76, 1.47
HVLT Delayed Recall score	0.85	0.62, 1.17	0.97	0.71, 1.31	0.79	0.57, 1.10	0.80	0.58, 1.09
Concurrent benzodiazepine	1.12	0.55, 2.27	0.81	0.41, 1.62	0.86	0.41, 1.80	0.73	0.35, 1.49
Concurrent atypical antipsychotic	1.45	0.72, 2.91	1.26	0.65, 2.44	1.02	0.50, 2.10	1.64	0.83, 3.27
Body mass index	1.31	0.93, 1.84	1.33	0.97, 1.83	1.63	1.15, 2.30	1.23	0.90, 1.70
History of suicide attempt	0.99	0.53, 1.86	1.02	0.55, 1.88	0.90	0.46, 1.74	1.00	0.53, 1.89
Inpatient status at first treatment	1.13	0.43, 2.95	1.05	0.41, 2.69	1.37	0.49, 3.87	1.27	0.47, 3.42
Presence of anxious features	0.84	0.43, 1.65	0.92	0.48, 1.76	0.53	0.26, 1.08	0.53	0.27, 1.05
Comorbid PTSD Diagnosis	1.62	0.70, 3.74	1.90	0.85, 4.25	1.55	0.69, 3.47	1.25	0.56, 2.77

© 2024 Jha MK et al. *JAMA Network Open*.

Footnotes: * same measures as the outcome measure. **OR expressed as per SD increase for continuous predictors and yes vs no for binary predictors. PTSD is post-traumatic stress disorder, QIDS-SR16 is 16-item Quick Inventory of Depressive Symptomatology Self-Report version, MADRS is Montgomery Åsberg Depression Rating Scale, MoCA is Montreal Cognitive Assessment, HVLT is Hopkins Verbal Learning Test, and NAART-35 is the North American Adult Reading Test 35.

© 2024 Jha MK et al. *JAMA Network Open*.
