# Supplementary material for: Ketamine vs Electroconvulsive Therapy for Treatment-Resistant Depression: A Secondary Analysis of a Randomized Clinical Trial
Source: JAMA Netw Open. 2024 Jun 25;7(6):e2417786. doi: 10.1001/jamanetworkopen.2024.17786 (PMC11200139; doi:10.1001/jamanetworkopen.2024.17786)
Supplement: Supplement 3. — Data Sharing Statement [file jamanetwopen-e2417786-s003.pdf]

Data Sharing Statement

Jha. Ketamine vs Electroconvulsive Therapy for Treatment-Resistant Depression. *JAMA Netw Open*. Published June 25, 2024. doi:10.1001/jamanetworkopen.2024.17786

Data
Data available: No
